# ILC1-derived IFN-γ regulates macrophage activation in colon cancer

**DOI:** 10.1186/s13062-023-00401-w

**Published:** 2023-09-07

**Authors:** Yandong Zhang, Shu Ma, Tie Li, Yu Tian, Huangao Zhou, Hongsheng Wang, Lan Huang

**Affiliations:** 1 Department of Rheumatology, The First Hospital of Jilin University, Changchun, People’s Republic of China; 2 Department of Laboratory Medicine, Suzhou Municipal Hospital, The Affiliated Suzhou Hospital of Nanjing Medical University, Suzhou, People’s Republic of China; 3 Department of emergency medicine, Jiangyin People’s Hospital, Wuxi, China; 4 Department of General Surgery, The Affiliated Hospital of Yangzhou University, Yangzhou, China

**Keywords:** Tumor-associated macrophage, M1 macrophage, Group 1 innate lymphocytes, Colon cancer, IFN-γ

## Abstract

**Background:**

Tumor-associated macrophages (TAMs) are an important subset of innate immune cells in the tumor microenvironment, and they are pivotal regulators of tumor-promoting inflammation and tumor progression. Evidence has proven that TAM numbers are substantially increased in cancers, and most of these TAMs are polarized toward the alternatively activated M2 phenotype; Thus, these TAMs strongly promote the progression of cancer diseases. Type 1 innate lymphocytes (ILC1s) are present in high numbers in intestinal tissues and are characterized by the expression of the transcription factor T-bet and the secretion of interferon (IFN)-γ, which can promote macrophages to polarize toward the classically activated antitumor M1 phenotype. However, the relationship between these two cell subsets in colon cancer remains unclear.

**Methods:**

Flow cytometry was used to determine the percentages of M1-like macrophages, M2-like macrophages and ILC1s in colon cancer tissues and paracancerous healthy colon tissues in the AOM/DSS-induced mouse model of colon cancer. Furthermore, ILC1s were isolated and bone marrow-derived macrophages were generated to analyze the crosstalk that occurred between these cells when cocultured in vitro. Moreover, ILC1s were adoptively transferred or inhibited in vivo to explore the effects of ILC1s on tumor-infiltrating macrophages and tumor growth.

**Results:**

We found that the percentages of M1-like macrophages and ILC1s were decreased in colon cancer tissues, and these populations were positively correlated. ILC1s promoted the polarization of macrophages toward the classically activated M1-like phenotype in vitro, and this effect could be blocked by an anti-IFN-γ antibody. The in vivo results showed that the administration of the Group 1 innate lymphocyte-blocking anti-NK1.1 antibody decreased the number of M1-like macrophages in the tumor tissues of MC38 tumor-bearing mice and promoted tumor growth, and adoptive transfer of ILC1s inhibited tumors and increased the percentage of M1-like macrophages in MC38 tumor-bearing mice.

**Conclusions:**

Our studies preliminarily prove for the first time that ILC1s promote the activation of M1-like macrophages by secreting IFN-γ and inhibit the progression of colon cancer, which may provide insight into immunotherapeutic approaches for colon cancer.

**Supplementary Information:**

The online version contains supplementary material available at 10.1186/s13062-023-00401-w.

## Introduction

Colon cancer is a malignant and lethal cancer disease that affects the gastrointestinal tract, and it is the second leading cause of tumor-related death in the world [1]. Currently, surgery combined with radiotherapy and chemotherapy is the most common treatment for colon cancer, although the therapeutic effect is not satisfactory [2]. To date, immunotherapy, which is a promising therapeutic strategy, has been used to treat a variety of tumor diseases [3, 4]. Understanding the mechanism by which the immune response is regulated during the occurrence and development of colon cancer is a key step in developing immunotherapeutic strategies to treat colon cancer.

Innate lymphocytes (ILCs), which are a subset of innate immune cells, participate in the regulation of multiple tumor diseases [5]. Studies have shown that ILCs are present in high numbers in colon cancer tissues and play an important role in the development of colon cancer [6]; However, the regulatory mechanism is not fully clear. ILCs are divided into three subsets according to the transcription factors they express and the cytokines they secrete: Group 1 innate lymphocytes, Group 2 innate lymphocytes, and Group 3 innate lymphocytes [7]. Among these subsets, Group 1 innate lymphocytes, which include conventional NK cells and ILC1s, are characterized by the secretion of interferon (IFN)-γ and tumor necrosis factor (TNF)-α, which exert powerful antitumor effects on various tumors [8]. TNF-α was proven to have a potent antitumor effect. TNF-α can not only kill tumors directly but also promote cell death and pyroptosis, thus indirectly recruiting and stimulating antitumor macrophages and dendritic cells (DCs) to promote antitumor responses. In addition, TNF-α is also required for the induction and activation of cytotoxic T cells (CTLs), which are the most effective tumor-killing cells [9, 10]. IFN-γ, which is a main effector factor of Group 1 innate lymphocytes, inhibits the proliferation and angiogenesis of tumor cells and enhances the cytotoxic activity of CTLs to promote antitumor effects [11, 12]. It is worth noting that IFN-γ induces the polarization of macrophages toward the classically activated M1-like phenotype and then promotes the tumor-inhibiting effect of these cells [13, 14]. However, it remains unclear whether Group 1 innate lymphocytes can promote M1-like macrophage polarization in colon cancer.

Tumor-associated macrophages (TAMs) are the main subset of myeloid cells that infiltrate solid tumors, and they play a key role in coordinating tumor-induced inflammation [15]. TAMs mainly include tumor-promoting M2-like macrophages in the tumor microenvironment, but in response to certain stimuli, TAMs can also polarize toward the antitumor M1-like phenotype. This functional heterogeneity of TAMs is common in a variety of tumors [16, 17]. IFN-γ and lipopolysaccharide (LPS) promote macrophage polarization toward the classically activated antitumor M1-like phenotype, while IL-4 and IL-13 result in macrophage polarization toward the M2-like phenotype and can be used as immunotherapeutic targets in macrophage-based tumor immunotherapy [18–20]. Studies have shown that the proportion of M2-like macrophages, which are characterized by CD206 and Arg-1 expression, is increased in the colon cancer microenvironment, while the proportion of M1-like macrophages, which are characterized by CD86 and iNOS expression, is decreased in the colon cancer microenvironment, leading to immunosuppressive and tumor-promoting effects in colon cancer [21, 22]. Therefore, regulating the M1/M2 balance is a promising approach to improve immunotherapeutic effects in colon cancer [23]. However, the mechanism by which M1-like macrophage numbers are decreased in the colon cancer microenvironment is still not fully understood.

## Materials and methods

### Mice

Male and female C57BL/6 mice (8 weeks, 20 g) were purchased from the Animal Center of Jilin University (Jilin, China). All mice were bred and maintained in a specific pathogen-free animal facility with a temperature of 23 °C ± 2 °C and relative humidity of 55% ± 10%. The mice were sacrificed by inhalation of carbon dioxide, and the mice were anesthetized with ketamine when the tumors were transplanted. All the animal experimental protocols were in accordance with the Guide for Care and Use of Laboratory Animals and approved by the Ethics Committee of Jilin University (20200698).

### Cell line and cell culture

The mouse MC38 colon cancer cell line and mouse RAW264.7 macrophage cell line were purchased from the Shanghai Cell Bank of the Chinese Academy of Sciences (Shanghai, China). Mouse bone marrow-derived macrophages (BMDMs) were generated from C57BL/6 mouse bone marrow, and mouse ILC1s were isolated from the spleens of C57BL/6 mice. The cells were cultured in DMEM or RPMI 1640 medium supplemented with 10% FBS. All the cells were cultured in a humidified incubator with 5% CO_2_ and at 37 °C.

### Isolation of type 1 innate lymphocytes

ILC1s were isolated from the spleens of C57BL/6 mice by combining magnetic beads and flow sorting. Mouse spleen cells were used to generated single-cell splenocyte suspensions[24]. The obtained cells were incubated with biotin-conjugated antibodies (anti-CD3ε, anti-CD45R, anti-Gr-1, anti-CD11c, anti-CD11b, anti-Ter119, anti-TCR-αβ, and anti-FCεRI; Miltenyi, Belgish, Germany) to enrich lineage-negative cells following the Miltenyi magnetic bead cell isolation protocol. Enriched lineage-negative single cell suspensions were stained with an anti-mouse lineage cocktail (BioLegend, 145-2C11, RB6-8C5, RA3-6B2, Ter-119, and M1/70), anti-mouse CD127 (BioLegend, A7R34), anti-mouse NK1.1 (BioLegend, PK136), and anti-mouse NKp46 (BioLegend, 29A1.4) for flow cytometry sorting (BD FACS Melody) of lineage^−^ CD127^+^ NK1.1^+^ NKp46^+^ cells (which were considered ILC1s); these cells were cultured in RPMI 1640 medium supplemented with 10% FBS and 0.5 ng/ml IL-7 (Gibco, PMC0071).

### Induction of bone marrow-derived macrophage

The legs of C57BL/6 mice (8 weeks, female) were harvested, and a syringe was used to collect bone marrow cells in a 10 cm cell culture plate on a clean bench. The bone marrow cells were washed three times, and the cells were passed through a 70 μm cell strainer into a 15 ml cell collection tube. The samples were centrifuged at 1000 rpm for 5 min, 5 ml of ACK was added to lyse the red blood cells, and then the samples were centrifuged at 1000 rpm for 5 min again. Bone marrow cells were cultured in 6-well plates with 4 ml culture medium (supplemented with 10% FBS and 20 ng/ml M-CSF) at a cell density of 1 × 10^6^ cells per well. On the third day, half of the culture medium was changed, and the corresponding stimulating factor, M-CSF, was added. On the fifth day, all of the culture medium was changed, and the corresponding stimulating factor, M-CSF, was added. On the seventh day, bone marrow-derived macrophages (BMDMs) were collected for the experiments, and the purity of the BMDMs was determined by flow cytometry (Additional file 1: Fig. S1B).

For the coculture of ILC1s and macrophages, we used RPMI 1640 medium supplemented with 10% FBS and 0.5 ng/ml IL-7. RAW264.7 cells or BMDMs were mixed with ILC1s and cocultured in 24-well plates for 24 h.

### Co-culture of cells

ILC1s and RAW264.7 or BMDMs were co-cultured in Transwell in 12-well plates at a 5:1 ratio, with ILC1s in the upper chamber and RAW264.7 or BMDMs in the lower chamber. IFN-γ and TNF-α neutralizing antibodies were added to the culture system at a concentration of 10 μg/mL. After 24 h, ILC1s were discarded, and the adherent RAW264.7 or BMDMs were collected for subsequent detection.

### Establishment of the mouse tumor model

To establish the AOM/DSS-induced mouse model of colitis-associated colon cancer, 8-week-old mice were intraperitoneally injected with azoxymethane (AOM, 10 mg/kg body weight) (Ray Biotech, Georgia, USA). Four days later, the mice were administered 1.5% dextran sodium sulfate (DSS) in their drinking water for six days. The DSS water was replaced with normal water for two weeks, and then the mice were treated with 1.5% DSS water for another 6 days. After 3 cycles of DSS treatment, nearly 3 months, tumor formation was evaluated. Once the tumor formation, we will sacrificed the mice and isolate the tumors, and we used paracancerous healthy colon tissues as control.

To establish the MC38 tumor-bearing mouse model, cultured MC38 cells were collected, and then the mice were subcutaneously injected via the right flank with 1 × 10^6^ tumor cells. And the tumor usually occurs at day 7–10, and we continued to monitor the tumor size. And we sacrificed the mice to analyze the tumor-infiltrating ILC1s and macrophage when tumor size has the obviously difference between different groups. Tumor length (L), diameter and width (W) were measured with a caliper.

### Flow cytometry analysis

Tumor tissues from MC38 tumor-bearing mice and colon cancer mice samples were isolated and cut into small pieces. Paracancer tissue as a control and digested as the tumor tissue. The tumor tissue pieces and paracancer tissue pieces were digested with digestion solution containing 5% FBS, collagenase II (1 mg/mL), DNase I (200 μg/mL) and hyaluronidase (4U/mL) (Sigma, St. Louis, USA) in 37 °C water bath for 30 min. 70 mesh-screen to collect digested single cells, centrifuge, and then obtain the single tumor tissue cell suspension. Anti-mouse CD45 (30-F11), anti-mouse F4/80 (BM8) were used to analyze macrophage, anti-mouse CD86 (A17199A) for M1-like phenotype macrophage, anti-mouse CD206 (15-2) for M2-like phenotype macrophage. anti-mouse Lineage (145-2C11), anti-mouse CD127 (A7R34), and anti-mouse T-bet (4B10) were used to detect ILC1s. For surface staining, antibody mixed with single cell suspension (100 µl/10^6^ cells) for 20 min at 4 degree. For intracellular staining, cells were cultured in complete 1640 medium with phorbol 12-myristate (PMA, 1ug/ml), ionomycin (1 µg/ml) and brefeldin A (10 μg/ml) (eBioscience, San Diego, USA) for 4–6 h. Collecting the cells, and then surface-stained, fixed, permeabilized. Using the Fixation and Permeabilization Buffer Kit (Thermo Fisher Scientific, Massachusetts, USA) to stain the intracellular factors following the manufacturer’s recommendations. Cell samples were detected by CytoFLEXS (Beckman coulter) and data was analyzed by Flowjo7.6.

### RNA isolation and quantitative of Real-time PCR

Total RNA was extracted by TRIZOL reagent (Thermo Scientific, Massachusetts, USA) according the manufacturer’s recommendations. cDNA was reverse transcribed from total RNA using the ReverTra Ace qPCR RT kit (Toyobo, Osaka, Japan). Real-time PCR was performed in duplicate using Bio-Rad SYBR Green Super Mix (TAKARA, Tokyo, Japan) according to the manufacturer's instructions. The primers were designed using Premier 5.0 software and synthesized by Shanghai Invitrogen. The mouse IFN-γ and β-actin primer sequences were as follows: IFN-γ, Forward (5′_3′), TGGCGGTGCTGAGCTACTGG; Reverse (5′_3′), TGTACCAGGAGTGTCAAGGCTCTC. β-actin, Forward (5′_3′), TGGAATCCTGTGGCATCCATGAAA; Reverse (5′_3′), TAAAACGCAGCTCAGTAACAGTCCG.

### Western blotting analysis

Cells were lysed with radioimmunoprecipitation assay (RIPA) buffer supplemented with a complete protease inhibiting tablet and then centrifuged to harvest the total proteins. The concentrations of the extracted protein samples were determined with a CBA kit. Equal amounts of total proteins were loaded into gels, separated by 10% SDS‒PAGE, and transferred to polyvinylidene fluoride (PVDF) membranes (Bio‐Rad, CA, USA). BSA (5%) was used to block nonspecific binding at room temperature for 2 h. The membranes were washed with PBS 1 time and then incubated with anti-iNOS and anti-Arg-1 primary antibodies and anti-β-actin antibodies (Abcam, Shanghai, China) overnight. The membranes were washed with TBS supplemented with 5% Tween 20 (TBST) 3 times for 10 min each. Then, the membranes were incubated with the secondary antibody at room temperature for 1 h. We cut the membranes before hybridization with antibodies during blotting to avoid wasting samples. A Luminescent Image Analyzer (Image Quant LAS4000mini, GE Healthcare, China) was used to detect the protein bands, and the density of the relevant bands was quantified by ImageJ.

### Statistical analysis

The data are presented as the mean ± SEM, and the data were analyzed by GraphPad Prism Version 7 software (GraphPad Software, CA, USA). Each in vitro experiment was performed in triplicate, and each in vivo experiment included 3–6 mice per treatment group. Statistical significance was determined by a t test and one-way ANOVA, and statistical significance was defined as a *p* value < 0.05.

## Results

### The macrophage population is increased in the colon cancer microenvironment and mainly includes CD206^+^ macrophages

**Fig. 1:**
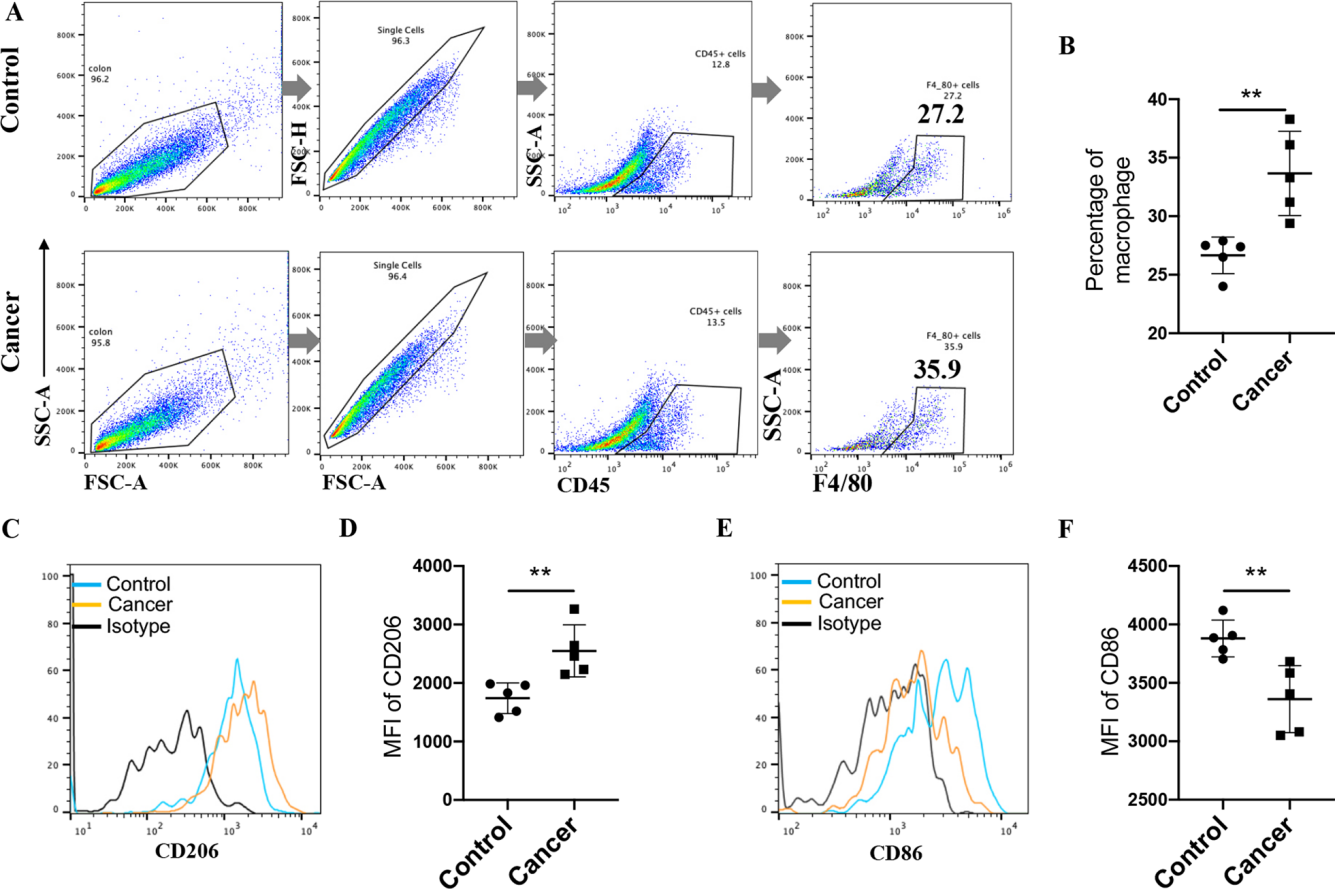
Macrophage CD206 expression is up-regulated in colon cancer tissue. **A** representative flow cytometry graphs of getting the colon cancer tissue and paracancer control tissue macrophage from AOM/DSS-induced mice colon cancer model, Fc-receptor-blocking antibody was used before staining the macrophage for flow cytometry analysis. **B** percentage of macrophage (CD45^+^F4/80^+^) in tumor tissue and control, n = 5. **C**, **D** CD206 mean fluorescence intensity (MFI) of macrophage (CD45^+^F4/80^+^CD206^+^) was detected by flow cytometry, n = 5. **E**, **F** CD86 MFI of macrophage (CD45^+^F4/80^+^CD86^+^) was detected by flow cytometry, n = 5. All the experiments repeated for 3 times. Data are shown as mean ± SD (n = 5 per group, *p < 0.05, **p < 0.01, ***p < 0.001, and *****p < 0.0001)

Tumor-infiltrating macrophages have always been considered important factors that regulate tumor development and progression, and in particular, the ratio of antitumor M1-like macrophages to tumor-promoting M2-like macrophages plays an important role in tumor regulation [25, 26]. To examine the role of macrophages in colon cancer, we established an AOM/DSS-induced mouse model of colon cancer and determined the percentages of tumor-infiltrating macrophages (CD45^+^ F4/80^+^), CD86 and CD206 expression of macrophages in mouse colon cancer tissues and paracancerous healthy colon tissues. An Fc-receptor-blocking antibody was applied before the macrophages were stained for flow cytometry analysis, and the flow cytometry results showed that the tumor-infiltrating macrophage population was increased in mouse colon cancer tissues compared to healthy tissues (Fig. 1A, B); This is consistent with previous studies showing that macrophage numbers increased in the colon cancer microenvironment when tumor occurred [27]. Flow cytometry results also proved that macrophage CD86 expression decreased while macrophage CD206 expression increased in the colon cancer microenvironment (Fig. 1C–F, Additional file 1: Fig. S1A), which demonstrated that macrophages were polarized toward the tumor-promoting M2-like phenotype in the colon cancer microenvironment to accelerate cancer progression. However, the mechanism by which the macrophage CD86 expression decreased and the macrophage CD206 expression increased in colon cancer is not fully understood.

### ILC1 numbers are decreased in the colon cancer microenvironment and are positively correlated with macrophage expressing CD86

**Fig. 2:**
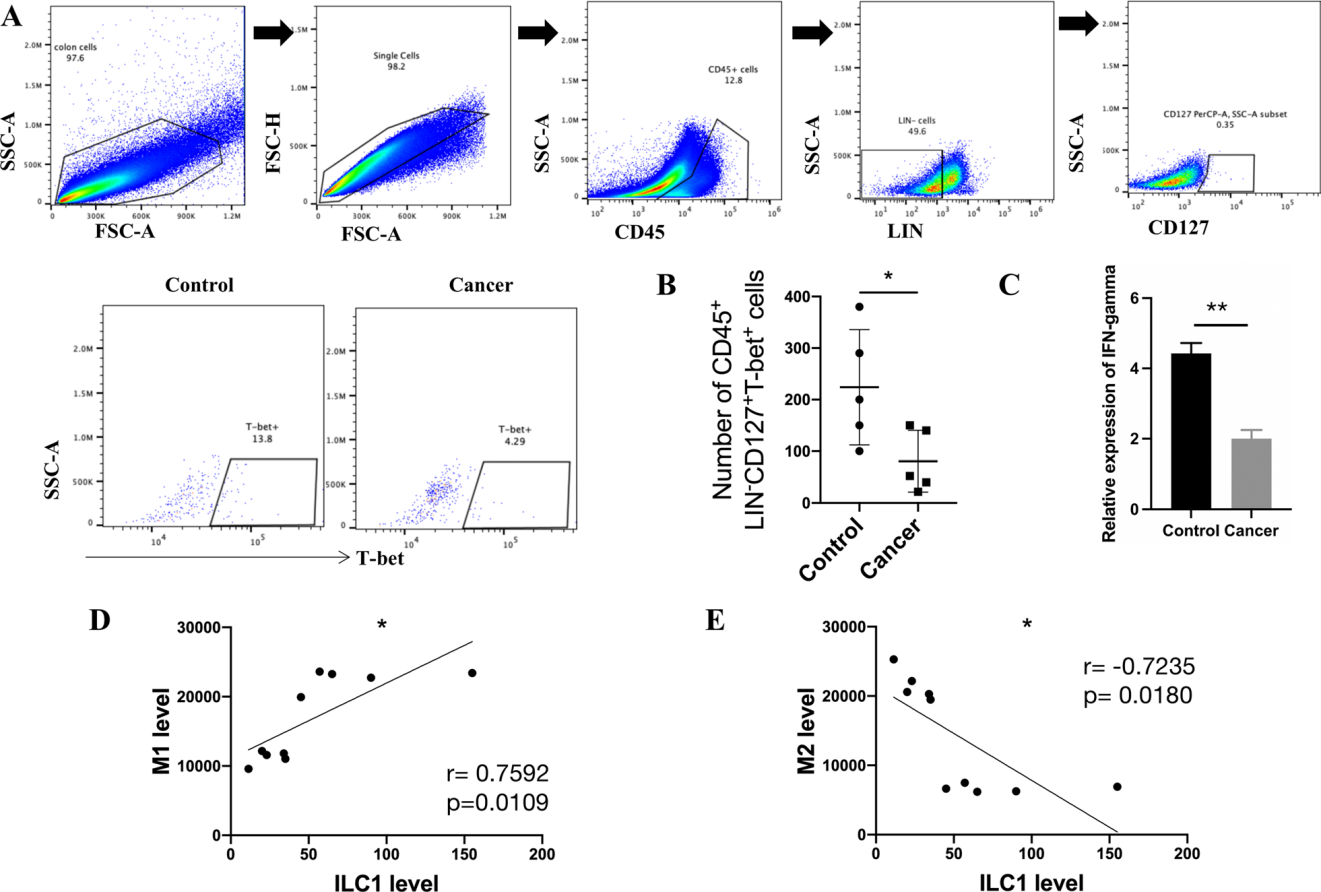
Percentage of ILC1s is positive correlation with the level of M1-like phenotype macrophage. **A** Representative flow cytometry graphs of ILC1s getting strategy in the colon cancer tissue and paracancer control tissue. **B** Relative number of ILC1s (CD45^+^Lineage^−^CD127^+^T-bet^+^) in tumor tissue and control, n = 5. **C** Relative expression of IFN-γ in cancer tissue and control was detected by RT-PCR. **D, E** The levels of tumor-infiltrated ILC1s compared to tumor-infiltrated macrophage CD86 MFI (M1 level) and macrophage CD206 MFI (M2 level) was made the regression analysis. All the experiments repeated for 3 times. Data are shown as mean ± SD (n = 5 per group, *p < 0.05, **p < 0.01, ***p < 0.001, and ***p < 0.0001)

Previous studies have shown that ILCs are present in high numbers in intestinal tissues, and ILC2-derived IL-4 can promote tumorigenesis by polarizing macrophages toward the pro-tumorigenic M2-like phenotype [28–30]. Furthermore, ILC1s are characterized by the secretion of IFN-γ, which can polarize macrophages toward the M1-like phenotype [31, 32]. On this basis, we hypothesized that the decreased proportion of macrophage CD86 expression (M1-like macrophages) occurred due to the downregulation of IFN-γ in the cancer microenvironment. We analyzed the percentage of ILC1s (CD45^+^ Lineage^−^ CD127^+^ T-bet^+^) in colon cancer tissues and paracancerous healthy colon tissues from AOM/DSS-induced model mice. Flow cytometry results showed decreased ILC1 numbers in mouse colon cancer tissues compared to healthy tissues (Fig. 2A, B, Additional file 1: Fig. S1C). RT‒PCR results also showed decreased expression of IFN-γ in mouse colon cancer tissues compared to healthy tissues (Fig. 2C). In addition, the macrophage CD86 expression was positively correlated with the ILC1 percentage in mouse colon cancer tissues, while the macrophage CD206 expression was negatively correlated with the ILC1 percentage. (Fig. 2D, E). Whether the decrease in M1-like macrophages contributes to the decrease in ILC1s in mouse colon cancer tissues remains unclear.

### ILC1s promote M1-like macrophage polarization in vitro

**Fig. 3:**
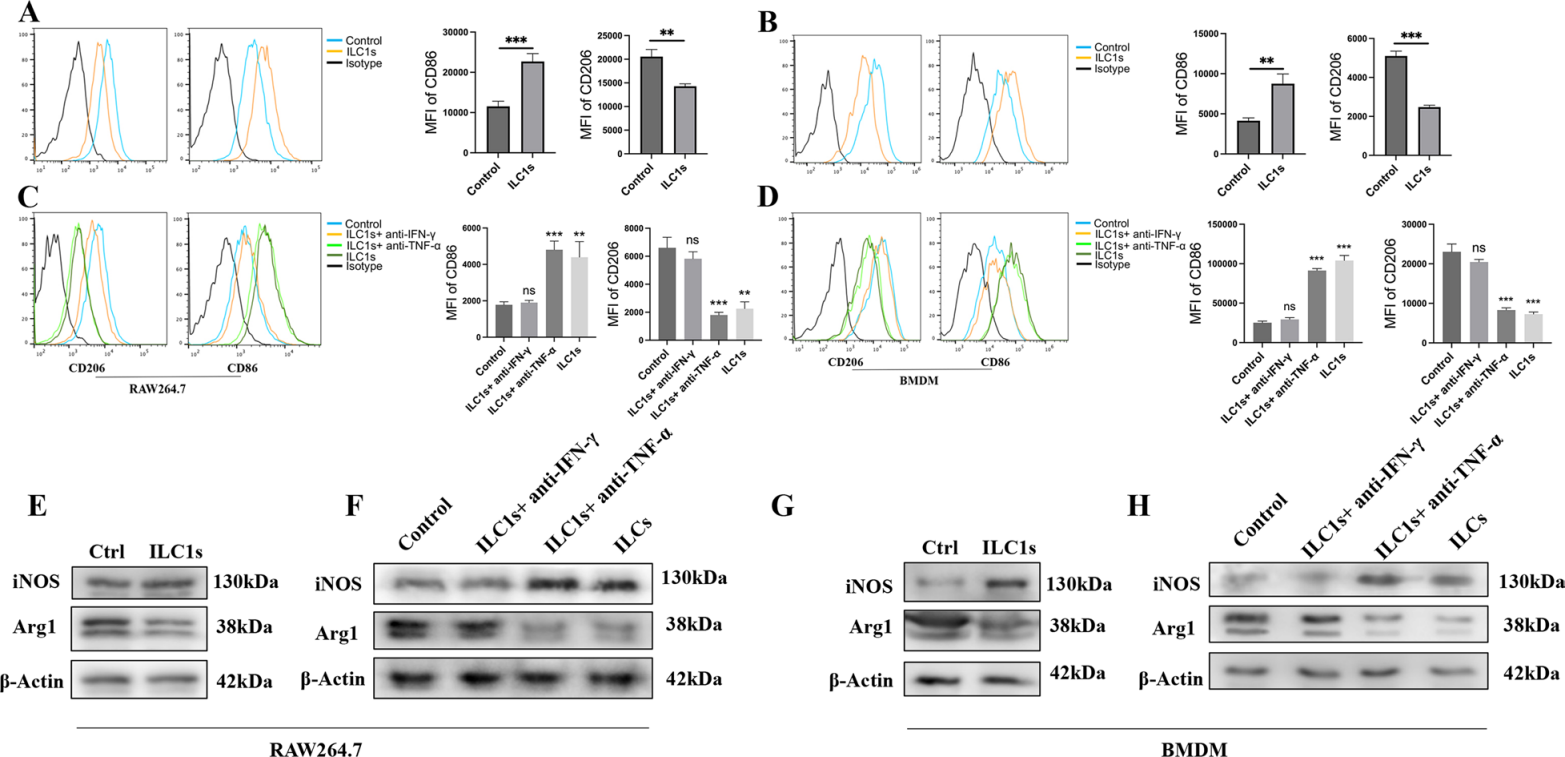
ILC1s promote M1-like phenotype macrophage polarizing in vitro. Sorted ILC1s (1 × 10^5^) from mouse spleen co-cultured with macrophage cell line RAW264.7 (2 × 10^4^) and bone marrow-derived macrophage (BMDM) for 24 h. **A** representative flow cytometry graphs and statistical chart of the expression of CD206 and CD86 in ILC1s co-cultured RAW264.7 and control RAW264.7. **B** representative flow cytometry graphs and statistical chart of the expression of CD206 and CD86 in ILC1s co-cultured BMDM and control BMDM. **C**, **D** 10 µg/ml anti-IFN-γ and TNF-α were added to co-culture system, flow cytometry was used to detected the expression of CD206 and CD86 in RAW264.7 and BMDM of different treated groups. **E**, **F** Western blotting was used to detect the expression of iNOS and Arg-1 in ILC1s co-cultured, anti-IFN-γ and TNF-α treated co-culture system, and control RAW264.7. **G**, **H** western blotting was used to detect the expression of iNOS and Arg-1 in ILC1s co-cultured, anti-IFN-γ and TNF-α treated co-culture system, and control BMDM. All the experiments repeated for 3 times

To prove that ILC1s could promote macrophage polarization toward the M1-like phenotype, we sorted ILC1s from mouse spleens and cocultured them with the RAW264.7 macrophage cell line or bone marrow-derived macrophages (BMDMs). Our results showed that CD86 was significantly upregulated on RAW264.7 cells and BMDMs that were cocultured with ILC1s, while CD206 expression was downregulated (Fig. 3A, B). To determine whether ILC1s-derived IFN-γ promotes M1 phenotype polarization, we chose to add neutralizing antibodies against the ILC1-derived factors IFN-γ and TNF-α to the coculture system. Flow cytometry results showed that anti-IFN-γ antibodies decreased the expression of CD86 on RAW264.7 cells and BMDMs, while anti-TNF-α antibodies failed to alter CD86 expression (Fig. 3C, D). The changes of iNOS and Arg1 protein levels in RAW264.7 (Fig. 3E, F) and BMDMs (Fig. 3G, H) co-cultured with ILC1s were also consistent with the results of FCM. Together, our results showed that ILC1s could promote macrophage polarization toward an M1-like phenotype by secreting IFN-γ in vitro.

### Adoptive transfer of ILC1s significantly decreases tumor growth in MC38 tumor-bearing mice

**Fig. 4:**
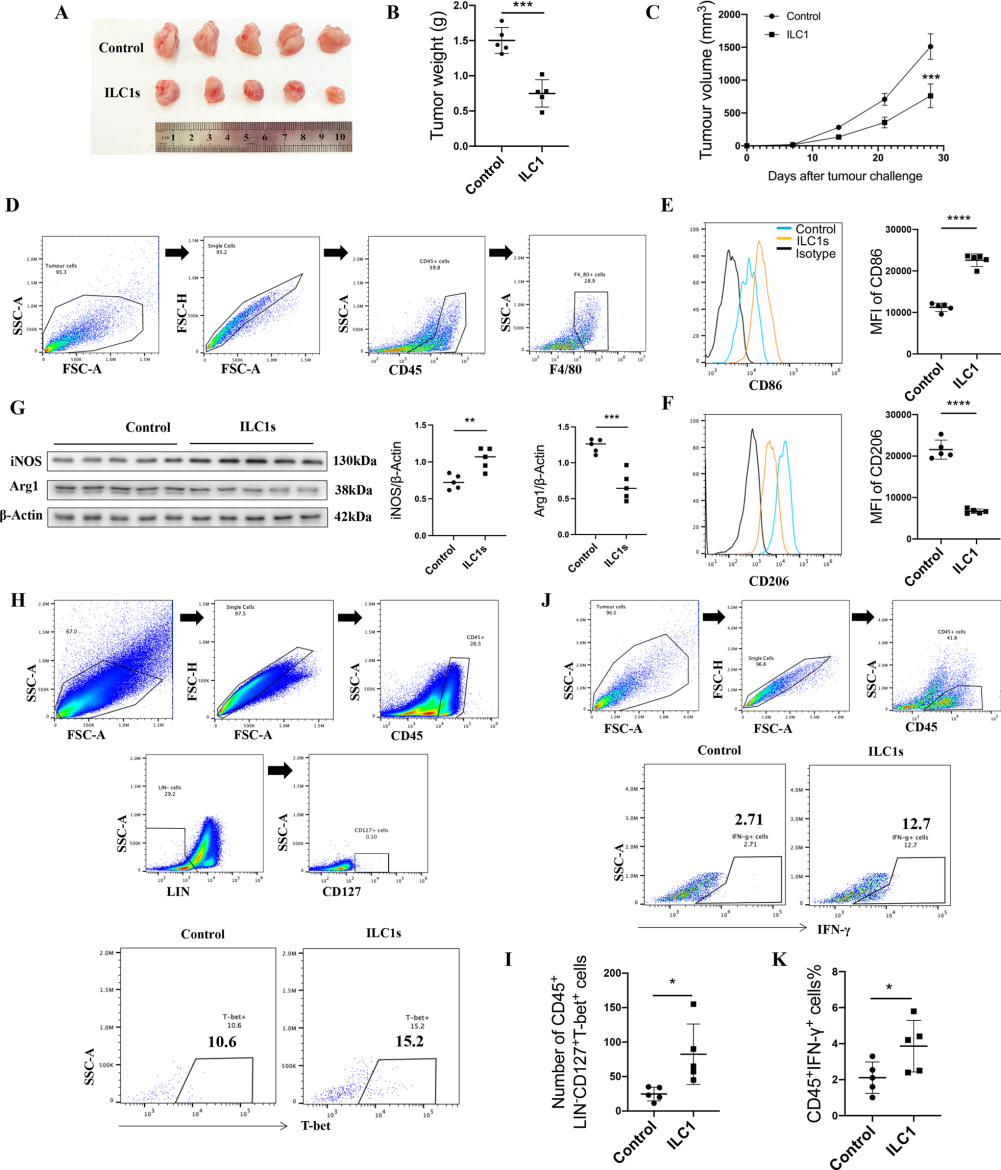
Adoptively transferred ILC1s inhibit the tumor growth by promoting the M1-like phenotype macrophage polarization. **A**–**C** the tumor graph, tumor weight, and tumor volume in 2 × 10^5^ ILC1s adoptively transferred mice and control mice. **D** Representative flow cytometry graphs of getting the tumor tissue macrophage. **E** CD86 MFI of macrophage (CD45^+^F4/80^+^CD86^+^) in the tumor of ILC1s adoptively transferred mice and control mice was detected by flow cytometry. **F** CD206 MFI of macrophage (CD45^+^F4/80^+^CD206^+^) in the tumor of ILC1s adoptively transferred mice and control mice was detected by flow cytometry. **G** Western blotting was used to detect the expression of iNOS and Arg-1 in the tumor of ILC1s adoptively transferred mice and control mice. **H** Representative flow cytometry graphs of getting the tumor tissue ILC1s in different groups. **I** Percentage of ILC1s in different groups. **J** Representative flow cytometry getting strategy of the tumor tissue IFN-γ in different groups. **K** The expression level of IFN-γ in the tumor of ILC1s adoptively transferred mice and control mice. All the experiments repeated for 3 times, n = 5. Data are shown as mean ± SD (n = 5 per group, *p < 0.05, **p < 0.01, ***p < 0.001, and ****p < 0.0001)

To determine whether ILC1s could regulate the polarization of tumor-infiltrating macrophages in order to participate in the progression of colon cancer, we isolated ILC1s from mouse spleens and adoptively transferred 2 × 10^5^ ILC1s into mice 3 days after the injection of MC38 cells into these mice. At the endpoint of this experiment, we found that the tumor size and weight of ILC1 recipient mice were significantly decreased compared to those of control mice (Fig. 4A–C). To prove that the difference in tumor growth between control and ILC1 recipient MC38 tumor-bearing mice is attributed to the phenotypic change in tumor-infiltrating macrophages, we then determine the percentages of M1-like and M2-like macrophages in the tumor tissues of the different groups. Flow cytometry results showed that the macrophage CD206 expression was significantly decreased after the adoptive transfer of ILC1s, while the macrophage CD86 expression was simultaneously increased (Fig. 4D–F). Considering the antitumor effects of M1-like macrophages, these results preliminarily explained the mechanism underling the tumor growth inhibition that was observed in ILC1 recipient mice. Western blotting results also proved this phenomenon (Fig. 4G). The in vitro experiments described above proved that ILC1s could promote macrophage polarization toward the antitumor M1-like phenotype by secreting IFN-γ. Therefore, we wondered whether this phenotypic change in macrophages occurs due to the increase in the proportion of ILC1s and the increased levels of IFN-γ that are secreted by these cells. Flow cytometry was used to determine the percentage of ILC1s and levels of IFN-γ in tumor tissues. The results showed that the percentage of ILC1s in the tumor microenvironment was significantly increased in ILC1 recipient mice, which preliminarily proved that adoptively transferred ILC1s reach the tumor microenvironment (Fig. 4H, I, Additional file 1: Fig. S1D). Flow cytometry results also proved that the levels of IFN-γ were upregulated in the tumors of ILC1 recipient mice (Fig. 4J, K). Overall, we preliminarily proved that ILC1s could promote macrophage polarization toward the M1-like phenotype to exert antitumor effects by secreting IFN-γ in vivo.

### Blocking ILC1s promotes the tumor growth of MC38 tumor-bearing mice by inhibiting M1-like macrophages

**Fig. 5:**
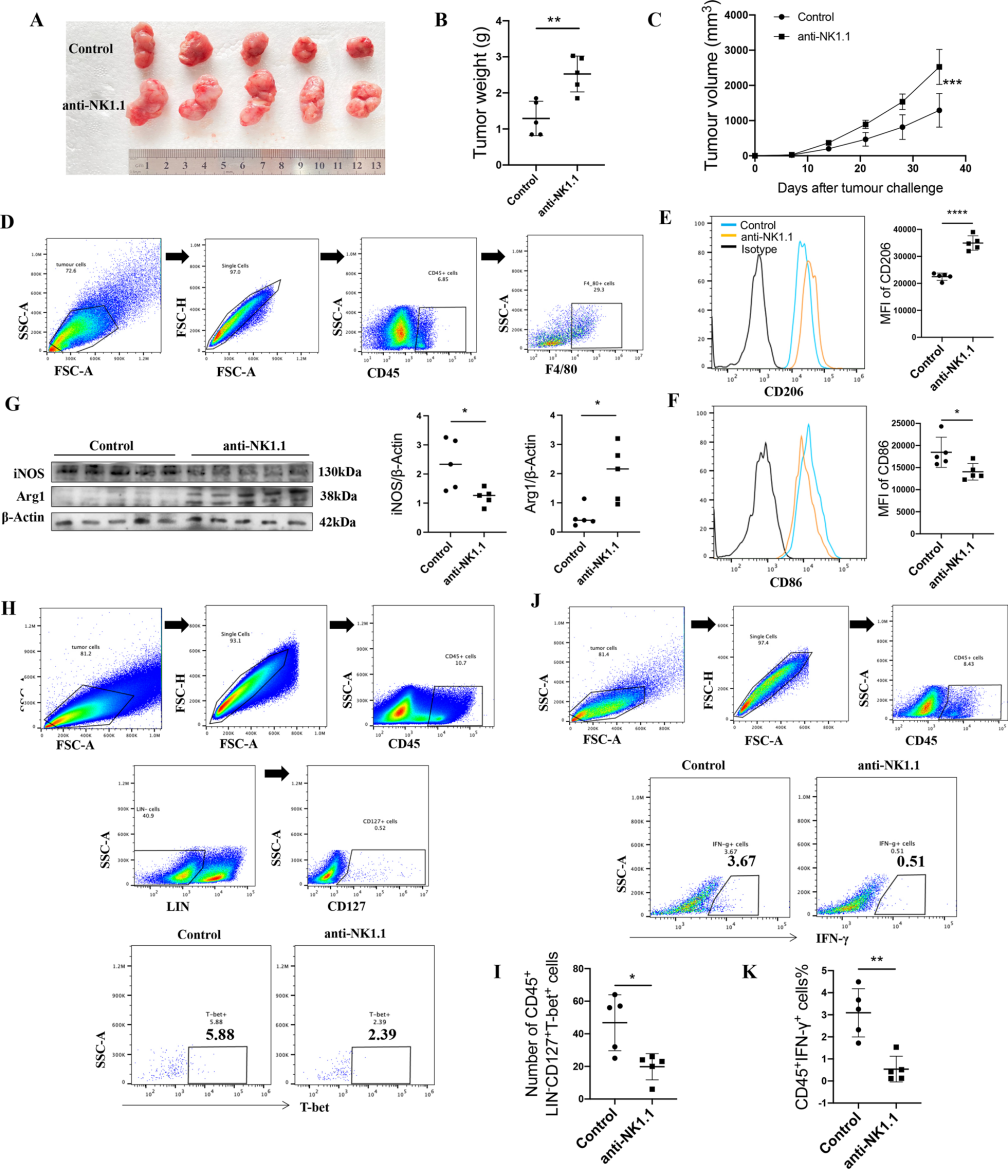
Blocking ILC1s promoting the tumor growth of MC38 tumor-bearing mice by inhibiting the M1-like phenotype macrophage polarization. **A–C** The tumor graph, tumor weight, and tumor volume in ILC1s mice and control mice, n = 5. **D** Representative flow cytometry graphs of getting the tumor tissue macrophage. **E** CD86 MFI of M1-like phenotype macrophage (CD45^+^F4/80^+^CD86^+^) in the tumor of ILC1s-blocking mice and control mice was detected by flow cytometry, n = 5. **F** CD206 MFI of M2-like phenotype macrophage (CD45^+^F4/80^+^CD206^+^) in the tumor of ILC1s mice and control mice was detected by flow cytometry, n = 5. **G** Western blotting was used to detect the expression of iNOS and Arg-1 in the tumor of ILC1s mice and control mice. **H** Representative flow cytometry graphs of getting the tumor tissue ILC1s in different groups. **I** Relative number of ILC1s in different groups, n = 5. **J** Representative flow cytometry graphs of getting the tumor tissue IFN-γ in different groups. **K** The expression level of IFN-γ in the tumor of ILC1s mice and control mice, n = 5. All the experiments repeated for 3 times. Data are shown as mean ± SD (n = 5 per group, *p < 0.05, **p < 0.01, ***p < 0.001, and ****p < 0.0001)

We further proved that ILC1s exert antitumor effects by restoring macrophages to an antitumorigenic M1-like phenotype. We performed an assay that involved ILC1s and a previously described Group 1 innate lymphocyte-neutralizing antibody, namely, anti-NK1.1 (PK136, Biolegend), in MC38 tumor-bearing mice [33]. Our results demonstrated that blocking ILC1s significantly promoted tumor growth in MC38 tumor-bearing mice compared to control mice (Fig. 5A–C), which directly proved the antitumor role of ILC1s in MC38 tumor-bearing mice.

To prove whether this effect relies on tumor-infiltrating macrophages, we determined the percentage of CD86^+^ and CD206^+^ macrophages in tumors of the different groups. Flow cytometry results showed that blocking ILC1s increased tumorigenesis macrophage (expressing high level CD206), meanwhile decreased anti-tumorigenesis macrophage (expressing high level CD86) (Fig. 5D–F). Western blotting results also proved this phenomenon (Fig. 5G). These results further proved that the antitumor role of ILC1s might partially rely on phenotypic changes in tumor-associated macrophages. To determine the reason for the macrophage phenotype change, we determined the percentage of ILC1s and the levels of IFN-γ in the tumors in the different groups. Flow cytometry results showed that the percentage of ILC1s and the level of IFN-γ were significantly decreased in the tumors of ILC1 recipient mice (Fig. 5H, J, I, K). Furthermore, we used anti-IFN-γ to treat tumor-bearing mice, and analyze the tumor-infiltrating macrophages. Results showed that anti-IFN-γ treatment greatly down-regulated the CD86 expression level of tumor-infiltrating macrophages while up-regulated the CD206 expression of tumor-infiltrating macrophages (Additional file 1: Figure S2). Together, these results further proved the antitumorigenic role of ILC1s and preliminarily demonstrated that ILC1s might promote macrophage phenotypic changes by secreting IFN-γ to exert antitumorigenic effects in vivo.

## Discussion

In this study, we explored the potential relationship between tumor-infiltrating ILC1s and TAMs. We found that the percentages of ILC1s and antitumorigenic M1-like macrophages were decreased in the colon cancer microenvironment, and rates of these decreases were positively correlated. In vitro results proved that ILC1s could promote macrophages to polarize to the classically activated M1-like phenotype, and this effect was reversed by anti-IFN-γ antibodies. This finding preliminarily demonstrated the potential relationship between ILC1s and tumor-infiltrating macrophages and partially explained the positive correlation between the decreased percentages of ILC1s and macrophage CD86 expression level. In vivo studies revealed that ILC1s indeed inhibited tumor growth and upregulated the percentage of macrophage CD86 expression level, while ILC1-blocking antibodies promoted tumor growth in MC38 tumor-bearing mice and decreased macrophage CD86 expression level. These results further proved that ILC1s inherently regulate tumor-infiltrating macrophages and enriched our knowledge about the antitumor effect of ILC1s. Altogether, our study preliminarily proved that ILC1s could promote the polarization of antitumor M1-like macrophages by secreting IFN-γ in the colon cancer microenvironment, thus inhibiting tumor growth. It is worth noting that anti-NK1.1 treatment not only depleted ILC1s but also depleted NK cells. Therefore, more research should be performed to confirm the exact cell subset that is responsible for the effect we observed. In our next project, we will solve this problem. Furthermore, in the ILC1s transfer assay, we do not find direct evidence that proved that the increased percentage of ILC1s in the tumor microenvironment is attributed to adoptive transfer, so the use of CD45 congenic mice and/or labeling the adoptively transferred ILC1s is needed to further prove our results in future work.

ILCs, which are a unique subset of lymphoid cells, have been widely studied in recent years; they do not express specific antigen receptors, but they participate in a variety of diseases, such as allergic diseases, autoimmune diseases and tumor diseases [34, 35]. Compared to CD4^+^ T cells, ILCs are divided into three subset: ILC1s express the transcription factor T-bet and are characterized by the secretion of IFN-γ and TNF-α [36, 37]; Group 2 innate lymphocytes express the transcription factors RORα and GATA3 and are characterized by the secretion of type II cytokines IL-4, IL-5, IL-9 and IL-13 [38, 39]; and Group 3 innate lymphocyte express the transcription factor RORγt and are characterized by the secretion of IL-17 and IL-22 [40]. Group 1 innate lymphocytes, such as Th1 cells, have been reported to play a significant role in intestinal inflammatory diseases [41, 42]. In recent years, a substantial number of studies have focused on the regulatory function of ILC1s in tumor diseases [43]. Numerous studies have shown that ILC1s exert a tumor-inhibiting effect by secreting the tumor-killing factors IFN-γ and TNF-α [44, 45]. Herein, we found a new mechanism by which ILC1s exert their antitumorigenic effects in colon cancer. We found that ILC1s promoted macrophage polarization toward the antitumor M1-like phenotype by secreting IFN-γ and then inhibiting tumor growth, which highlights a new antitumor role of ILC1s. However, our study also has some limitations in that we only clearly proved that ILC1s could promote the induction of M1-like macrophages by secreting IFN-γ in vitro*,* and our in vivo results do not show that ILC1s directly affect tumor-infiltrating macrophages by secreting IFN-γ to perform an antitumor function in the tumor microenvironment. We only preliminarily proved this phenomenon, and more evidence is needed to further confirm these findings.

The tumor microenvironment (TME) is an abstract space where tumors grow and invade, and it is composed of tumor cells, interstitial cells, immune cells, capillaries, intercellular substances, tissue fluid and biomolecules [46]. The TME is a highly immunosuppressive environment, which is why tumors can evade the host’s immune surveillance and exhibit uncontrolled growth [47, 48]. Studies have proven that incompetent and exhausted effector T cells [49], myeloid-derived suppressor cells (MDSCs) [50], TAMs [51], regulatory T cells [52] and a variety of immunosuppressive factors are the main elements that form the immunosuppressive microenvironment of the TME [53]. Among these elements, TAMs play an especially critical role because they are the main immune cells that infiltrate the tumor microenvironment [54]. TAMs can recognize and eliminate tumor cells, but as tumors occur and develop, TAMs are polarized toward the M2 phenotype and play a role in promoting tumor growth, invasion and metastasis [55]. TAMs function as a "double-edged sword" in the occurrence and development of tumors. Determining why TAMs play dual roles, that is, determining why TAMs polarize toward the antitumor M1 phenotype or the tumor-promoting M2 phenotype, is a key step in the development of macrophage-based tumor immunotherapy. Studies have proven that an immunosuppressive TME always promotes macrophage polarization toward the M2 phenotype, which greatly further exacerbates tumor invasion and growth. Here, we found that the proportion of M2 phenotype macrophages was greatly increased in colon tumor tissues compared to healthy tissues and that these TAMs promoted tumor growth. Reversing M1 vs M2 polarization via the adoptive transfer of ILC1s greatly increased the percentage of M1 macrophages and inhibited tumor growth, which directly proved that the M1/M2 macrophage ratio is important for the development of tumors. Thus, identifying ways to increase the percentage of M1 macrophages and decrease the percentage of M2 macrophages is an important task for the further development of tumor immunotherapy.

## Conclusions

Our studies found that decreased ILC1s level might contribute to increased tumor-promoting macrophages (expressing high level CD206). ILC1s could promote the polarization of macrophage to highly CD86 expressing through secreting IFN-γ. In vivo studies demonstrated that adoptively transferred ILC1s increased the macrophage CD86 expressing and inhibited the tumor growth, while depletion of ILC1s reached the opposite. Our study may provide an insight in the immunotherapy of colon cancer.

### Supplementary Information

**Additional file 1**. Gating strategies.

**Additional file 2**. Anti-IFN-g down-regulates M1 macrophage while increase M2 Macrophage percentage.

**Additional file 3**. WB raw data.

**Additional file 4**. WB raw data.

## Data Availability

The data used to support the findings of this study are available in the supplemental data.

## Abbreviations

TAM: Tumor-associated macrophage
ILC1s: Type 1 innate lymphocytes
IFN-γ: Interferon-γ
ILCs: Innate lymphocytes
TNF-α: Tumor necrosis factor-α
DCs: Dendritic cells
CTL: Cytotoxic T cells
BMDM: Bone marrow-derived macrophage
TME: Tumor microenvironment
MDSCs: Myeloid-derived suppressor cells

## References

[1] Wen J, Min X, Shen M, Hua Q, Han Y, Zhao L, Liu L, Huang G, Liu J, Zhao X (2019). ACLY facilitates colon cancer cell metastasis by CTNNB1. J Exp Clin Cancer Res: CR.

[2] Otani K, Kawai K, Hata K, Tanaka T, Nishikawa T, Sasaki K, Kaneko M, Murono K, Emoto S, Nozawa H (2019). Colon cancer with perforation. Surg Today.

[3] Yang Y (2015). Cancer immunotherapy: harnessing the immune system to battle cancer. J Clin Investig.

[4] Kouidhi S, Ben Ayed F, Benammar Elgaaied A (2018). Targeting tumor metabolism: a new challenge to improve immunotherapy. Front Immunol.

[5] Wagner M, Koyasu S (2019). Cancer immunoediting by innate lymphoid cells. Trends Immunol.

[6] Wang S, Qu Y, Xia P, Chen Y, Zhu X, Zhang J, Wang G, Tian Y, Ying J, Fan Z (2020). Transdifferentiation of tumor infiltrating innate lymphoid cells during progression of colorectal cancer. Cell Res.

[7] Blom B, van Hoeven V, Hazenberg MD (2019). ILCs in hematologic malignancies: tumor cell killers and tissue healers. Semin Immunol.

[8] Chiossone L, Dumas PY, Vienne M, Vivier E (2018). Natural killer cells and other innate lymphoid cells in cancer. Nat Rev Immunol.

[9] Balkwill F (2009). Tumour necrosis factor and cancer. Nat Rev Cancer.

[10] Aggarwal BB, Gupta SC, Kim JH (2012). Historical perspectives on tumor necrosis factor and its superfamily: 25 years later, a golden journey. Blood.

[11] Gao Y, Yang J, Cai Y, Fu S, Zhang N, Fu X, Li L (2018). IFN-γ-mediated inhibition of lung cancer correlates with PD-L1 expression and is regulated by PI3K-AKT signaling. Int J Cancer.

[12] Hirata A, Hashimoto H, Shibasaki C, Narumi K, Aoki K (2019). Intratumoral IFN-α gene delivery reduces tumor-infiltrating regulatory T cells through the downregulation of tumor CCL17 expression. Cancer Gene Ther.

[13] Orecchioni M, Ghosheh Y, Pramod AB, Ley K (2019) Macrophage polarization: different gene signatures in M1(LPS+) vs. classically and M2(LPS-) vs. alternatively activated macrophages. Front Immunol 2019;10:1084. 10.3389/fimmu.2019.01084 PMC6543837 31178859

[14] Mezouar S, Mege JL (2020). Changing the paradigm of IFN-γ at the interface between innate and adaptive immunity: Macrophage-derived IFN-γ. J Leukoc Biol.

[15] Yin M, Shen J, Yu S, Fei J, Zhu X, Zhao J, Zhai L, Sadhukhan A, Zhou J (2019). Tumor-associated macrophages (TAMs): a critical activator in ovarian cancer metastasis. Onco Targets Ther.

[16] Ge Z, Ding S (2020). The crosstalk between tumor-associated macrophages (TAMs) and tumor cells and the corresponding targeted therapy. Front Oncol.

[17] Pan Y, Yu Y, Wang X, Zhang T (2020). Tumor-associated macrophages in tumor immunity. Front Immunol.

[18] Pervin M, Karim MR, Kuramochi M, Izawa T, Kuwamura M, Yamate J (2018). Macrophage populations and expression of regulatory inflammatory factors in hepatic macrophage-depleted rat livers under lipopolysaccharide (LPS) treatment. Toxicol Pathol.

[19] Zhang W, Zhang Y, He Y, Wang X, Fang Q (2019). Lipopolysaccharide mediates time-dependent macrophage M1/M2 polarization through the Tim-3/Galectin-9 signalling pathway. Exp Cell Res.

[20] Gao S, Zhou J, Liu N, Wang L, Gao Q, Wu Y, Zhao Q, Liu P, Wang S, Liu Y (2015). Curcumin induces M2 macrophage polarization by secretion IL-4 and/or IL-13. J Mol Cell Cardiol.

[21] Zhang L, Li Z, Skrzypczynska KM, Fang Q, Zhang W, O'Brien SA, He Y, Wang L, Zhang Q, Kim A (2020). Single-cell analyses inform mechanisms of myeloid-targeted therapies in colon cancer. Cell.

[22] Yahaya MAF, Lila MAM, Ismail S, Zainol M, Afizan N (2019). Tumour-associated macrophages (TAMs) in colon cancer and how to reeducate them. J Immunol Res.

[23] Zhang N, Liu C, Jin L, Zhang R, Wang T, Wang Q, Chen J, Yang F, Siebert HC, Zheng X (2020). Ketogenic diet elicits antitumor properties through inducing oxidative stress, inhibiting MMP-9 expression, and rebalancing M1/M2 tumor-associated macrophage phenotype in a mouse model of colon cancer. J Agric Food Chem.

[24] Wang Y, Yin K, Tian J, Xia X, Ma J, Tang X, Xu H, Wang S. Granulocytic myeloid-derived suppressor cells promote the stemness of colorectal cancer cells through Exosomal S100A9. Advanced Sci. (Weinheim, Baden-Wurttemberg, Germany) 2019;6(18):1901278. 10.1002/advs.201901278 PMC6755519 31559140

[25] Mantovani A, Sozzani S, Locati M, Allavena P, Sica A (2002). Macrophage polarization: tumor-associated macrophages as a paradigm for polarized M2 mononuclear phagocytes. Trends Immunol.

[26] Goswami KK, Ghosh T, Ghosh S, Sarkar M, Bose A, Baral R (2017). Tumor promoting role of anti-tumor macrophages in tumor microenvironment. Cell Immunol.

[27] Fang M, Li Y, Huang K, Qi S, Zhang J, Zgodzinski W, Majewski M, Wallner G, Gozdz S, Macek P (2017). IL33 promotes colon cancer cell stemness via JNK activation and macrophage recruitment. Can Res.

[28] Rao A, Strauss O, Kokkinou E, Bruchard M, Tripathi KP, Schlums H, Carrasco A, Mazzurana L, Konya V, Villablanca EJ (2020). Cytokines regulate the antigen-presenting characteristics of human circulating and tissue-resident intestinal ILCs. Nat Commun.

[29] Schneider C, Lee J, Koga S, Ricardo-Gonzalez RR, Nussbaum JC, Smith LK, Villeda SA, Liang HE, Locksley RM (2019). Tissue-resident group 2 innate lymphoid cells differentiate by layered ontogeny and in situ perinatal priming. Immunity.

[30] Wan J, Cai W, Wang H, Cheng J, Su Z, Wang S, Xu H (2020). Role of type 2 innate lymphoid cell and its related cytokines in tumor immunity. J Cell Physiol.

[31] Nabekura T, Riggan L, Hildreth AD, O'Sullivan TE, Shibuya A (2020). Type 1 innate lymphoid cells protect mice from acute liver injury via interferon-γ secretion for upregulating Bcl-xL expression in hepatocytes. Immunity.

[32] Vogel DY, Glim JE, Stavenuiter AW, Breur M, Heijnen P, Amor S, Dijkstra CD, Beelen RH (2014). Human macrophage polarization in vitro: maturation and activation methods compared. Immunobiology.

[33] Wu C, He S, Liu J, Wang B, Lin J, Duan Y, Gao X, Li D (2018). Type 1 innate lymphoid cell aggravation of atherosclerosis is mediated through TLR4. Scand J Immunol.

[34] Yuan X, Rasul F, Nashan B, Sun C. Innate lymphoid cells and cancer: role in tumor progression and inhibition. Eur J Immunol. 2021. 10.1002/eji.202049033 PMC8457100 34189723

[35] Poonpanichakul T, Chan-In W, Opasawatchai A, Loison F, Matangkasombut O, Charoensawan V, Matangkasombut P (2021). Innate lymphoid cells activation and transcriptomic changes in response to human dengue infection. Front Immunol.

[36] Ducimetière L, Lucchiari G, Litscher G, Nater M, Heeb L, Nuñez NG, Wyss L, Burri D, Vermeer M, Gschwend J et al. Conventional NK cells and tissue-resident ILC1s join forces to control liver metastasis. Proc Natl Acad Sci USA. 2021;118(27). 10.1073/pnas.2026271118 PMC8271692 34183415

[37] Bai L, Vienne M, Tang L, Kerdiles Y, Etiennot M, Escalière B, Galluso J, Wei H, Sun R, Vivier E, et al. Liver type 1 innate lymphoid cells develop locally via an interferon-γ-dependent loop. Science (New York, NY) 2021;371(6536). 10.1126/science.aba4177 33766856

[38] Shen C, Liu C, Zhang Z, Ping Y, Shao J, Tian Y, Yu W, Qin G, Liu S, Wang L (2021). PD-1 affects the immunosuppressive function of group 2 innate lymphoid cells in human non-small cell lung cancer. Front Immunol.

[39] Srinivasan A, Bajana S, Pankow A, Yuen C, Shah RK, Sun XH (2021). Type 2 innate lymphoid cells from Id1 transgenic mice alleviate skin manifestations of graft-versus-host disease. BMC Immunol.

[40] Chang Y, Kim JW, Yang S, Chung DH, Ko JS, Moon JS, Kim HY (2021). Increased GM-CSF-producing NCR(-) ILC3s and neutrophils in the intestinal mucosa exacerbate inflammatory bowel disease. Clin Translat Immunol.

[41] Clark JT, Christian DA, Gullicksrud JA, Perry JA, Park J, Jacquet M, Tarrant JC, Radaelli E, Silver J, Hunter CA. IL-33 promotes innate lymphoid cell-dependent IFN-γ production required for innate immunity to Toxoplasma gondii. eLife 2021, 10. 10.7554/eLife.65614 PMC8121546 33929319

[42] Castleman MJ, Dillon SM, Purba C, Cogswell AC, McCarter M, Barker E, Wilson C (2020). Enteric bacteria induce IFNγ and Granzyme B from human colonic Group 1 Innate Lymphoid Cells. Gut microbes.

[43] Moreno-Nieves UY, Tay JK, Saumyaa S, Horowitz NB, Shin JH, Mohammad IA, Luca B, Mundy DC, Gulati GS, Bedi N, et al. Landscape of innate lymphoid cells in human head and neck cancer reveals divergent NK cell states in the tumor microenvironment. Proc Natl Acad Sci USA 2021;118(28). 10.1073/pnas.2101169118 PMC8285950 34244432

[44] Nabekura T, Shibuya A (2021). Type 1 innate lymphoid cells: soldiers at the front line of immunity. Biomed J.

[45] Luci C, Bihl F, Bourdely P, Khou S, Popa A, Meghraoui-Kheddar A, Vermeulen O, Elaldi R, Poissonnet G, Sudaka A et al. Cutaneous squamous cell carcinoma development is associated with a temporal infiltration of ILC1 and NK cells with immune dysfunctions. J Investig Dermatol. 2021. 10.1016/j.jid.2021.03.018 33831432

[46] Bianchi G, Czarnecki PG, Ho M, Roccaro AM, Sacco A, Kawano Y, Gullà A, Aktas Samur A, Chen T, Wen K (2021). ROBO1 promotes homing, dissemination, and survival of multiple myeloma within the bone marrow microenvironment. Blood Cancer Discov.

[47] Russell BL, Sooklal SA, Malindisa ST, Daka LJ, Ntwasa M (2021). The tumor microenvironment factors that promote resistance to immune checkpoint blockade therapy. Front Oncol.

[48] Raghav K, Liu S, Overman MJ, Willett AF, Knafl M, Fu SC, Malpica A, Prasad S, Royal RE, Scally CP, et al. Efficacy, safety and biomarker analysis of combined PD-L1 (Atezolizumab) and VEGF (Bevacizumab) blockade in advanced malignant peritoneal mesothelioma. Cancer Discov. 2021. 10.1158/2159-8290.CD-21-0331 PMC8563380 34261675

[49] Gabriel SS, Tsui C, Chisanga D, Weber F, Llano-León M, Gubser PM, Bartholin L, Souza-Fonseca-Guimaraes F, Huntington ND, Shi W, et al. Transforming growth factor-β-regulated mTOR activity preserves cellular metabolism to maintain long-term T cell responses in chronic infection. Immunity 2021. 10.1016/j.immuni.2021.06.007 34233154

[50] Imazeki H, Ogiwara Y, Kawamura M, Boku N, Kudo-Saito C. CD11b(+)CTLA4(+) myeloid cells are a key driver of tumor evasion in colorectal cancer. J Immunother Cancer 2021;9(7). 10.1136/jitc-2021-002841 PMC8280900 34261702

[51] Wang Y, Miao Z, Qin X, Li B, Han Y. NOD2 deficiency confers a pro-tumorigenic macrophage phenotype to promote lung adenocarcinoma progression. J Cell Mol Med. 2021. 10.1111/jcmm.16790 PMC8335701 34268854

[52] Liu H, Yu Z, Tang B, Miao S, Qin C, Li Y, Liang Z, Shi Y, Zhang Y, Wang Q (2021). LYG1 deficiency attenuates the severity of acute graft-versus-host disease via skewing allogeneic T cells polarization towards treg cells. Front Immunol.

[53] Trebska-McGowan K, Chaib M, Alvarez MA, Kansal R, Pingili AK, Shibata D, Makowski L, Glazer ES. TGF-β alters the proportion of infiltrating immune cells in a pancreatic ductal adenocarcinoma. J Gastrointest Surg. 2021. 10.1007/s11605-021-05087-x PMC12289350 34260016

[54] Yu S, Li Q, Wang Y, Cui Y, Yu Y, Li W, Liu F, Liu T. Tumor-derived LIF promotes chemoresistance via activating tumor-associated macrophages in gastric cancers. Exp Cell Res. 2021;112734. 10.1016/j.yexcr.2021.112734 34265288

[55] Wang X, Ji Y, Feng P, Liu R, Li G, Zheng J, Xue Y, Wei Y, Ji C, Chen D, et al. The m6A reader IGF2BP2 regulates macrophage phenotypic activation and inflammatory diseases by stabilizing TSC1 and PPARγ. Adv Sci. (Weinheim, Baden-Wurttemberg, Germany) 2021;8(13):2100209. 10.1002/advs.202100209 PMC8261491 34258163

