# Correction: ILC1-derived IFN-γ regulates macrophage activation in colon cancer

**DOI:** 10.1186/s13062-023-00432-3

**Published:** 2023-12-04

**Authors:** Yandong Zhang, Shu Ma, Tie Li, Yu Tian, Huangao Zhou, Hongsheng Wang, Lan Huang

**Affiliations:** 1 Department of Rheumatology, The First Hospital of Jilin University, Changchun, People’s Republic of China; 2 Department of Laboratory Medicine, Suzhou Municipal Hospital, The Affiliated Suzhou Hospital of Nanjing Medical University, Suzhou, People’s Republic of China; 3 Department of emergency medicine, Jiangyin People’s Hospital, Wuxi, China; 4 Department of General Surgery, The Affiliated Hospital of Yangzhou University, Yangzhou, China

After publication of this article [1], it was brought to our attention that the first author’s name Yangon Zhang is incorrect, the correct name is Yan**dong** Zhang.

The original publication has been corrected.
