# Supplementary figures and images for: ILC1-derived IFN-γ regulates macrophage activation in colon cancer
Source: Biol Direct. 2023 Sep 7;18:56. doi: 10.1186/s13062-023-00401-w (PMC10486120; doi:10.1186/s13062-023-00401-w)

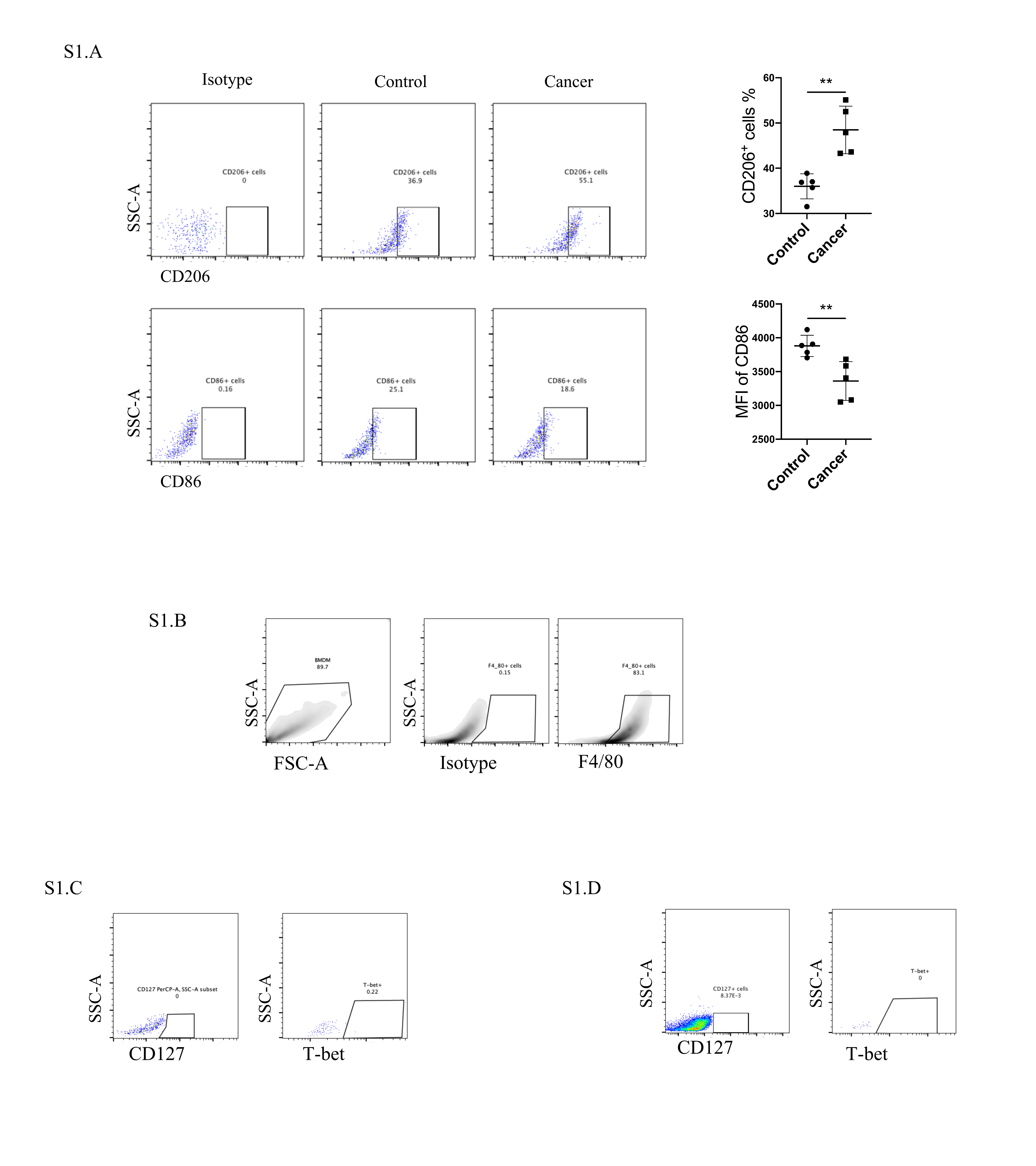

Supplement: Supplementary file 1 — Additional file 1. Gating strategies. [file 13062_2023_401_MOESM1_ESM.jpg]

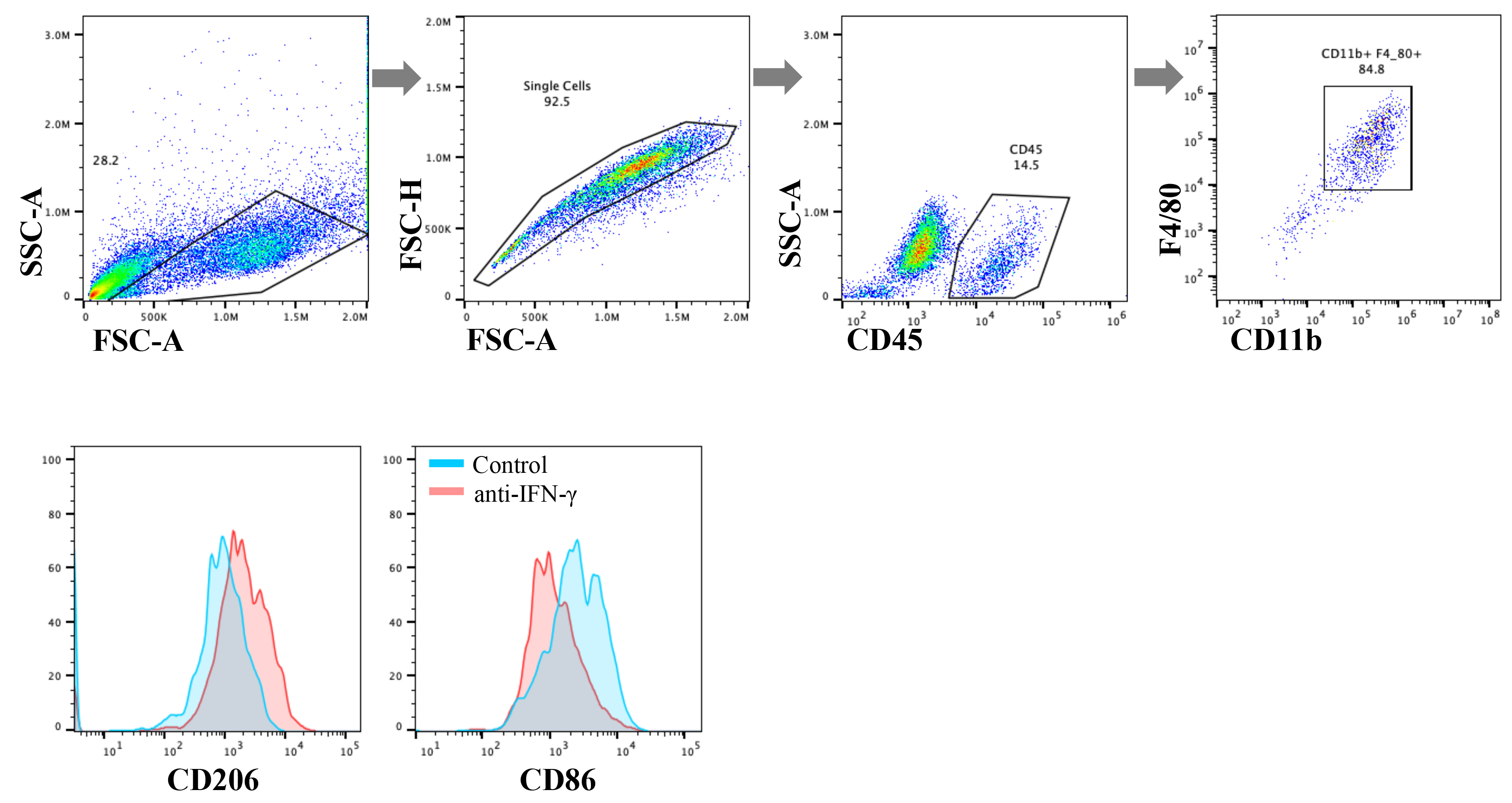

Supplement: Supplementary file 2 — Additional file 2. Anti-IFN-g down-regulates M1 macrophage while increase M2 Macrophage percentage. [file 13062_2023_401_MOESM2_ESM.jpg]

Fig 3E

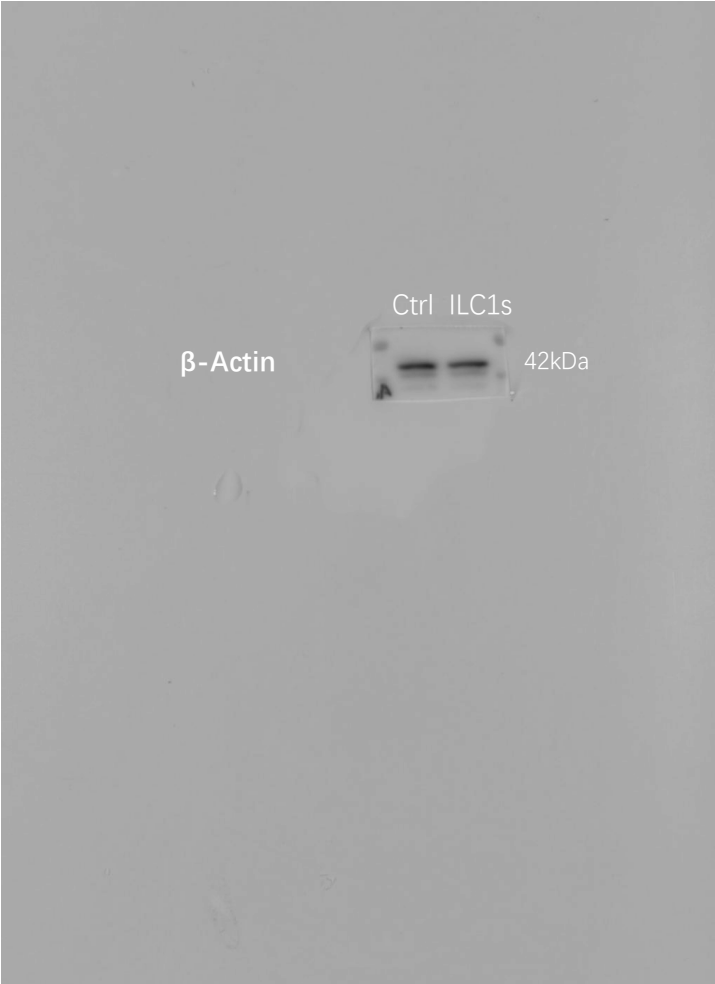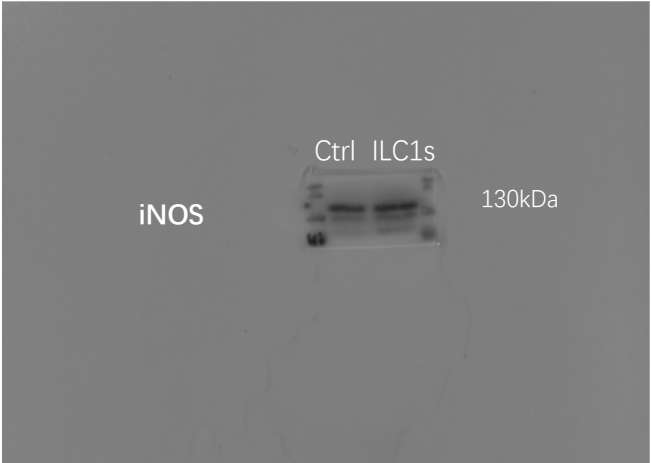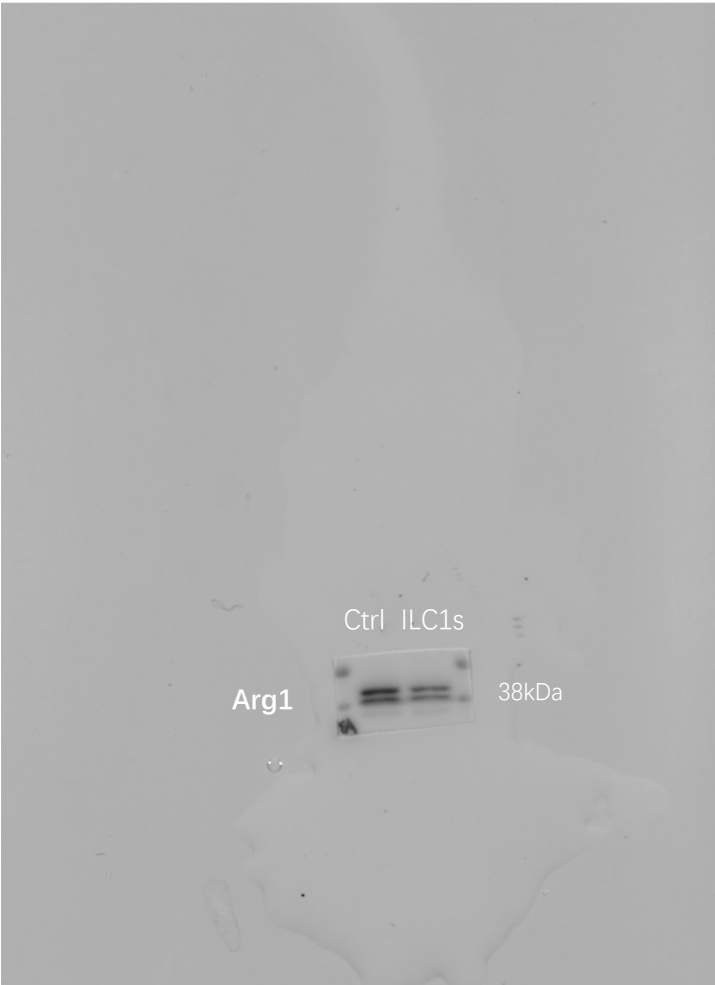

**Fig 3F**

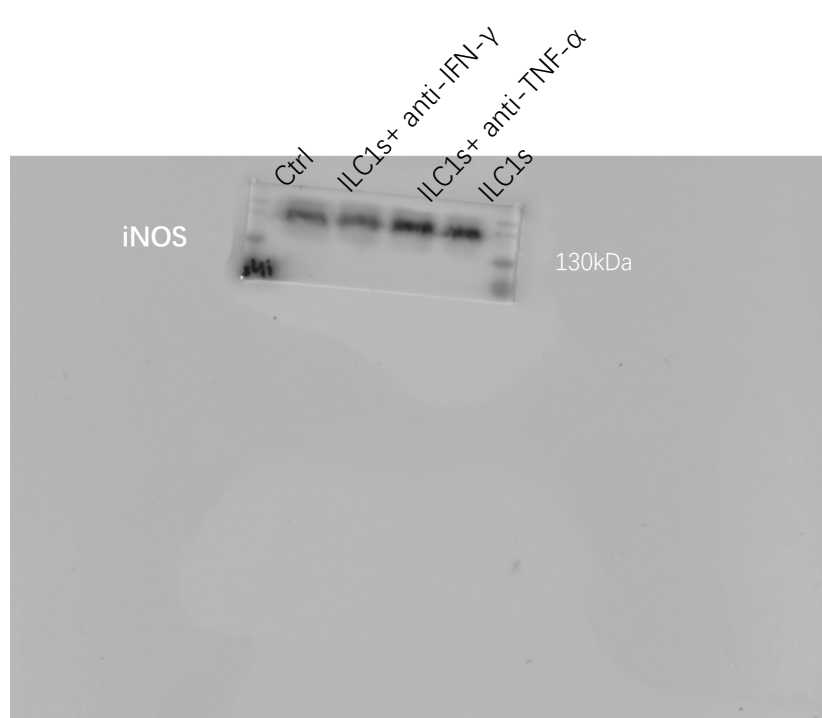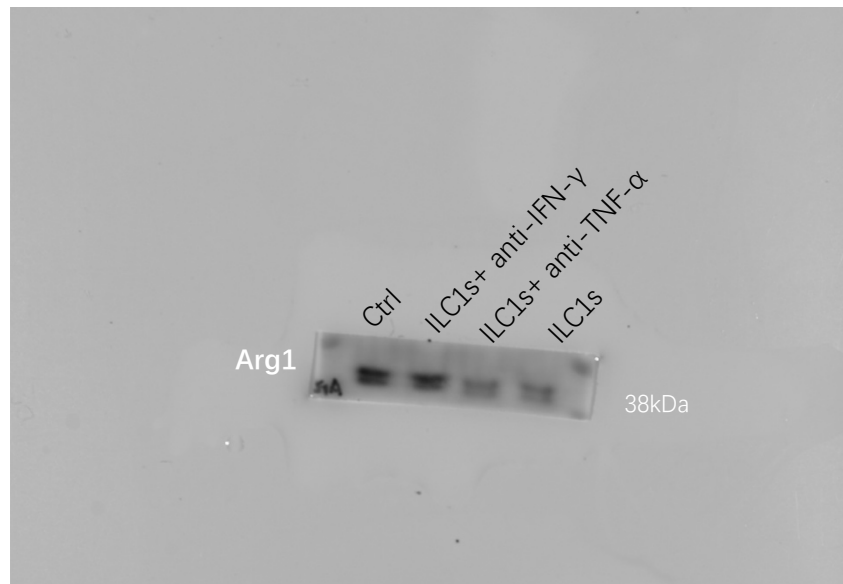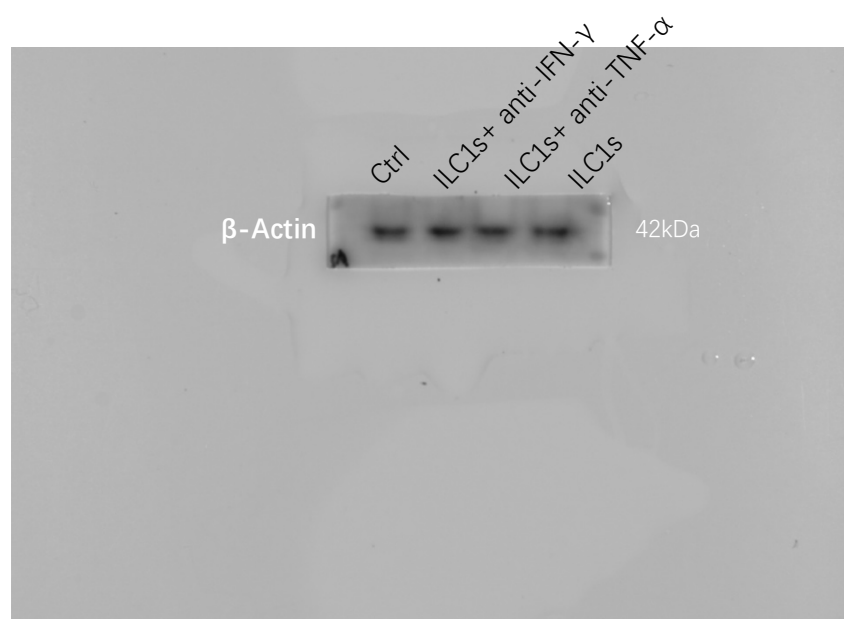

Fig 3G

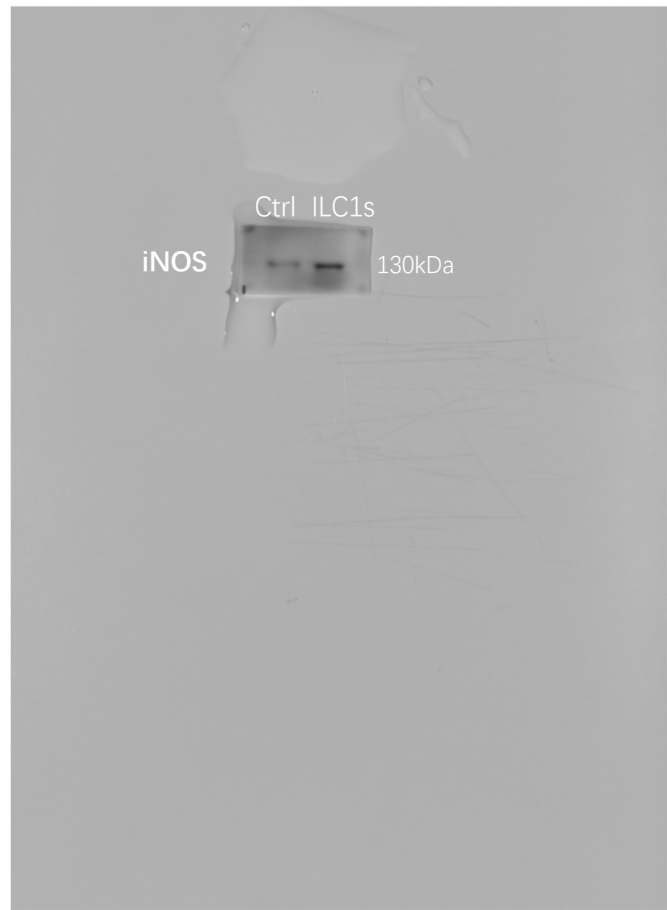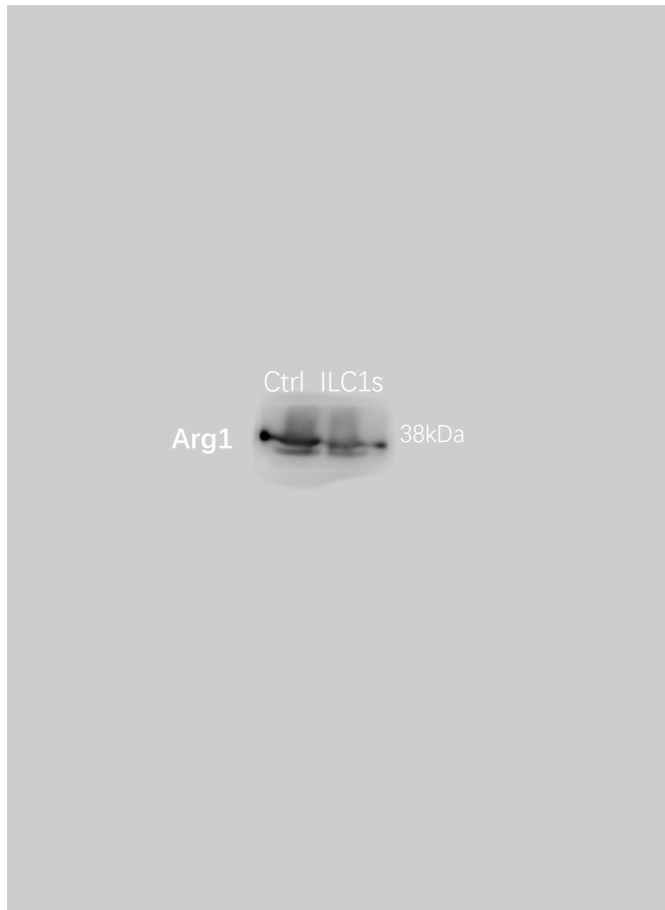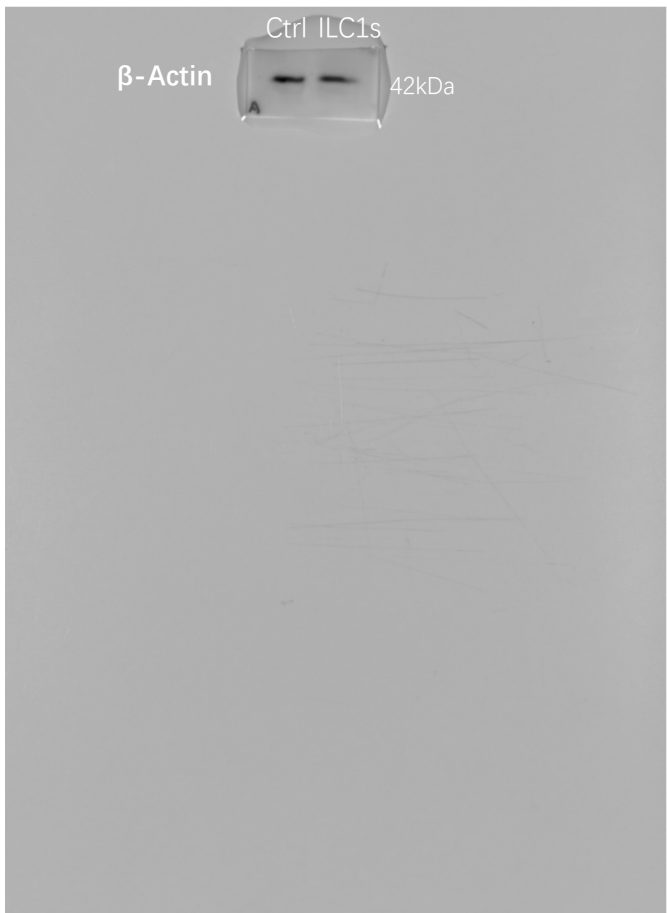

Fig 3H

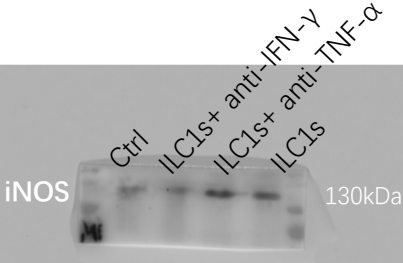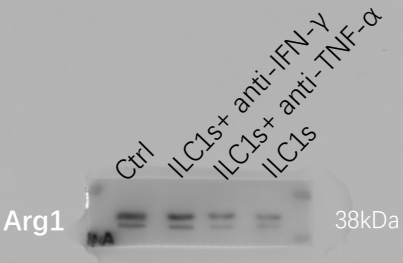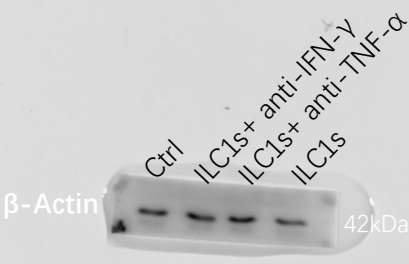

Fig 4G

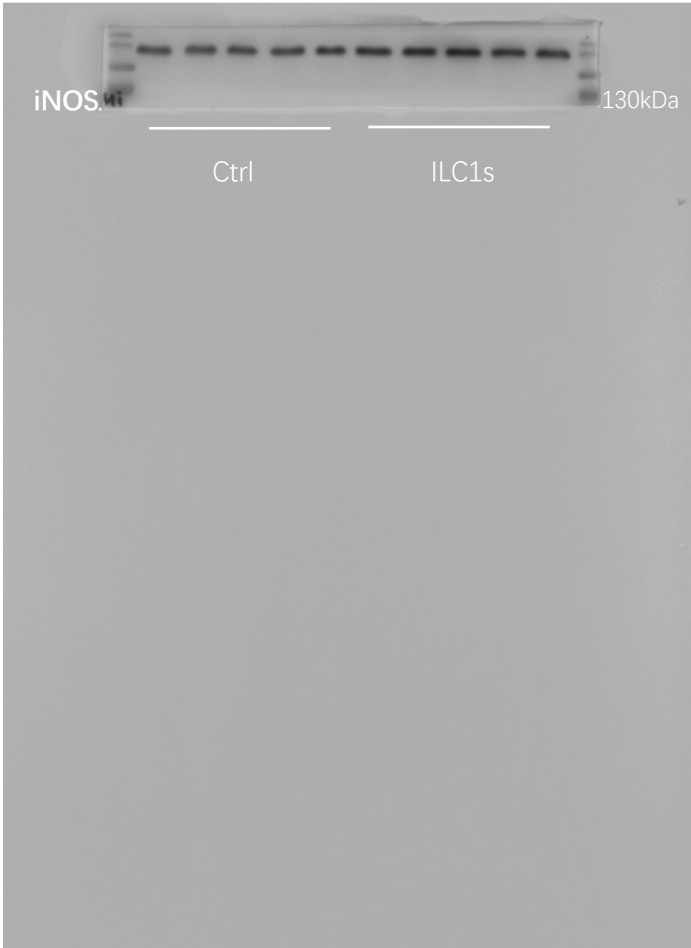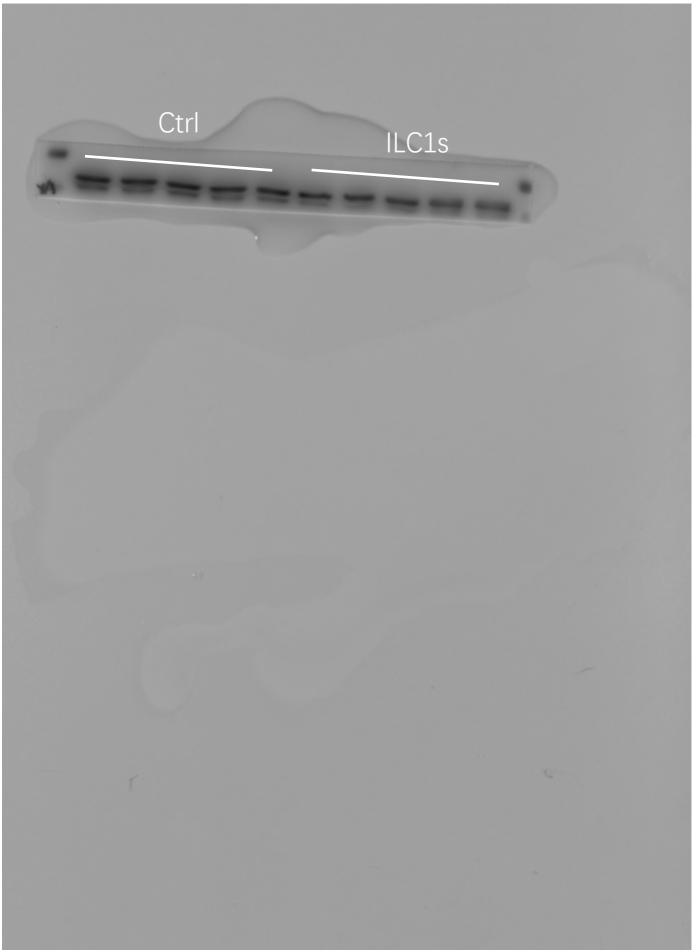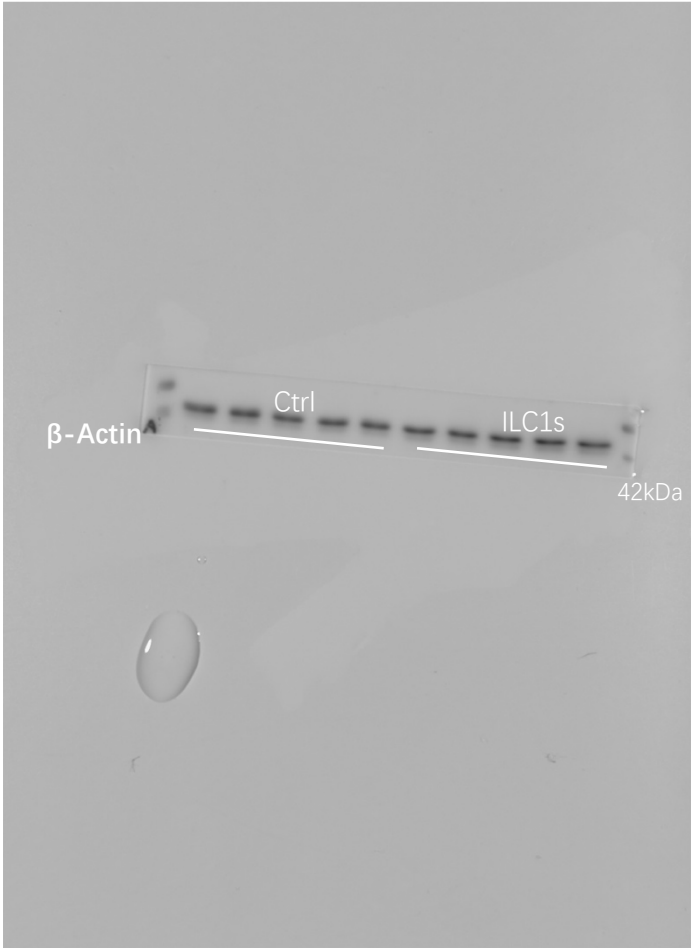

Fig 5G

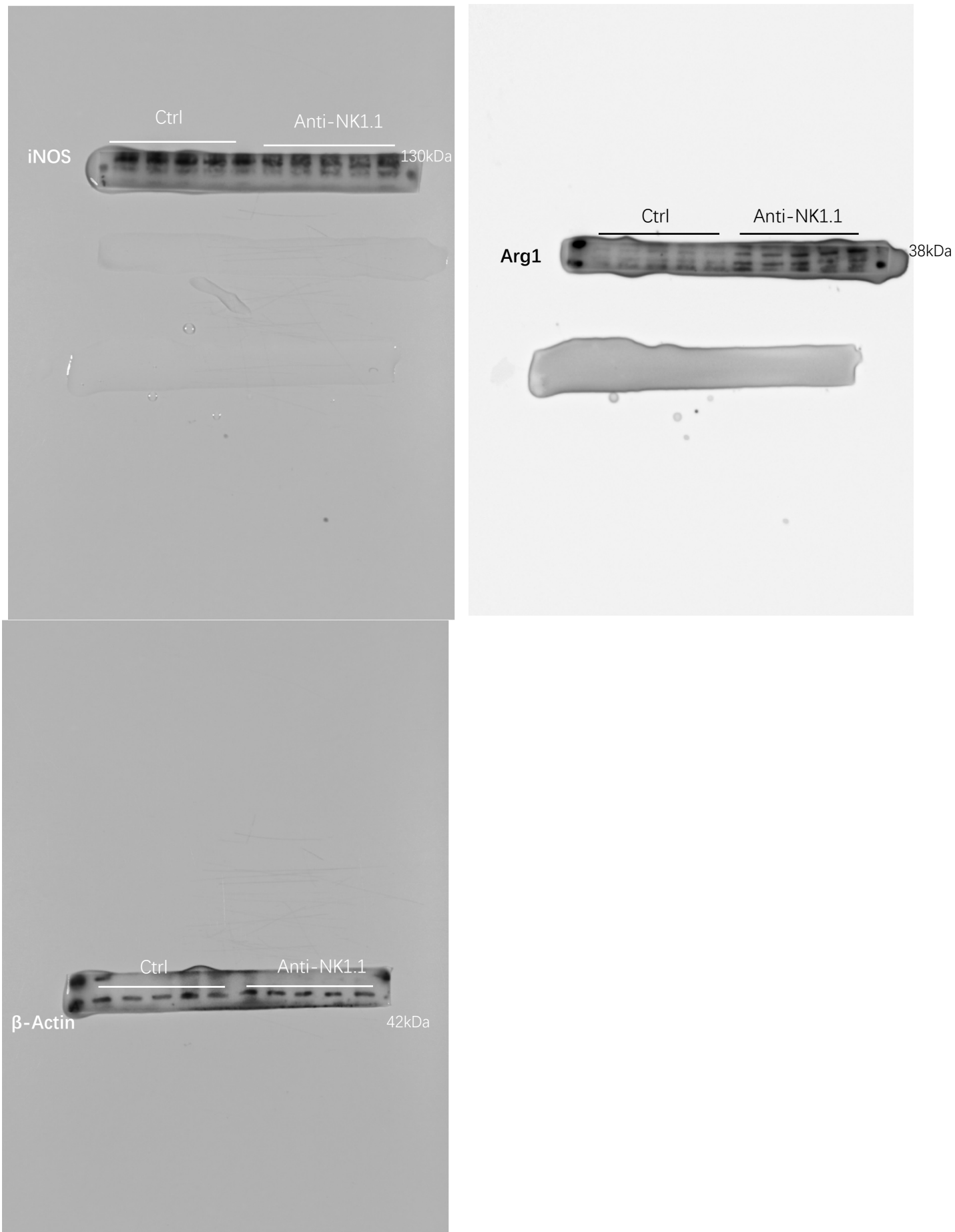

Supplement: Supplementary file 3 — Additional file 3. WB raw data. [file 13062_2023_401_MOESM3_ESM.pdf]

Fig3.E

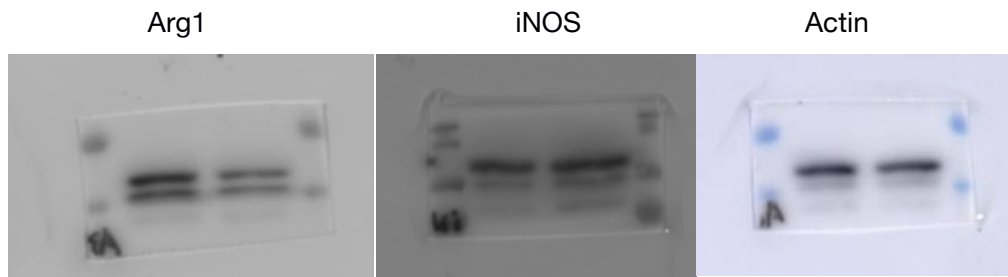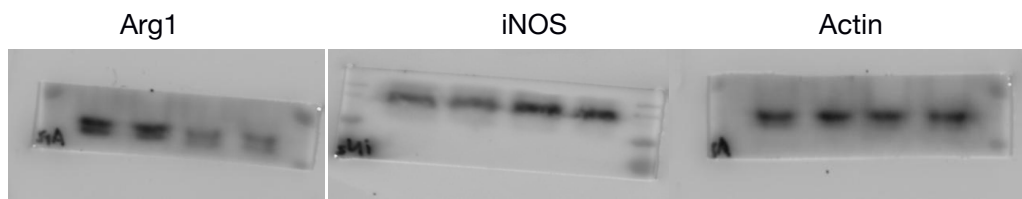

Fig 3.F

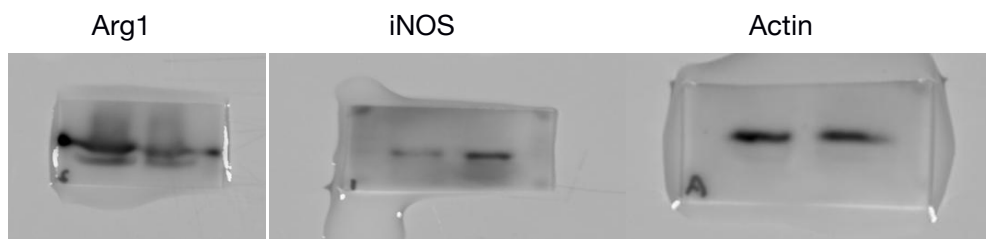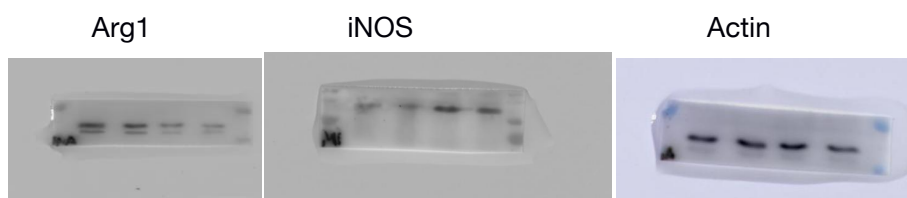

Fig4. G

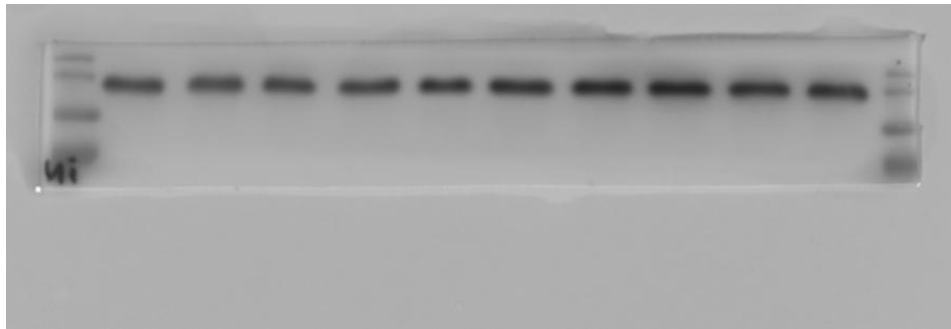

iNOS

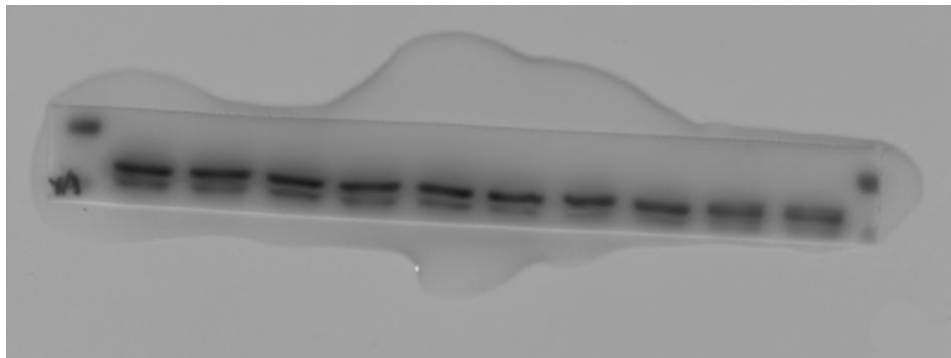

Arg-1

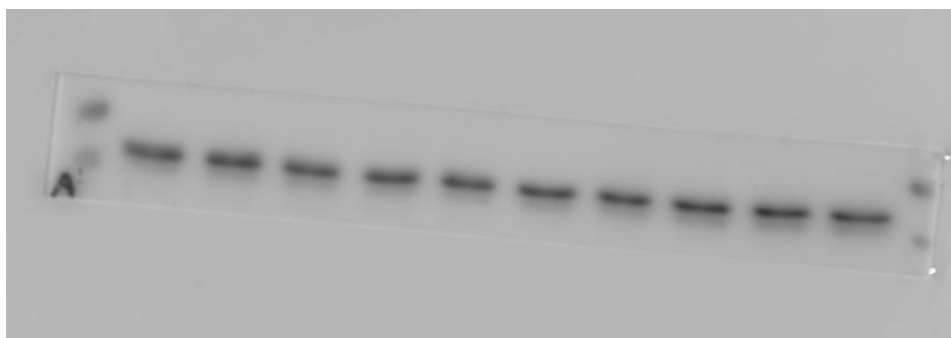

Actin

Fig 5G

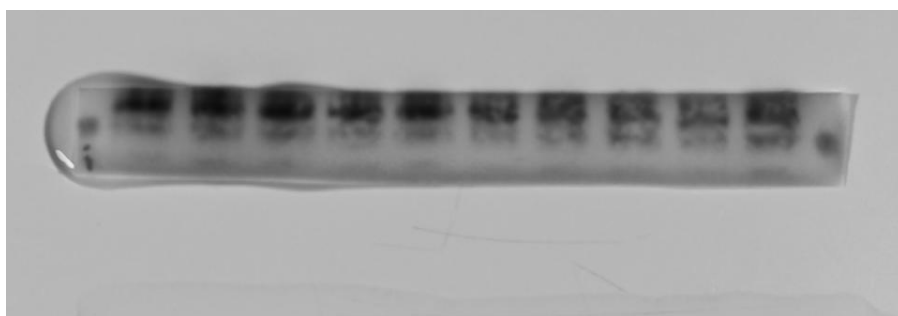

iNOS

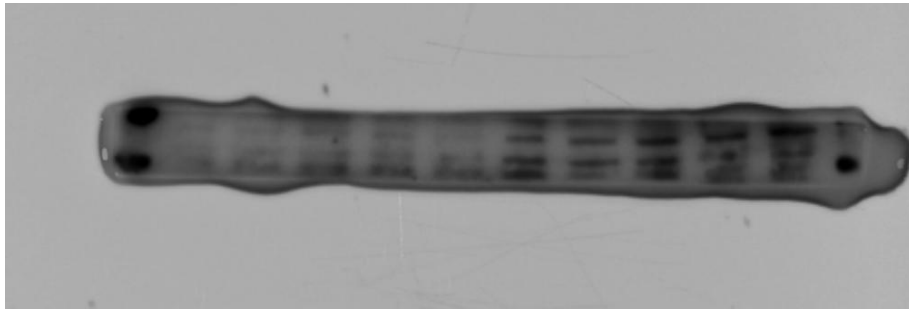

Arg1

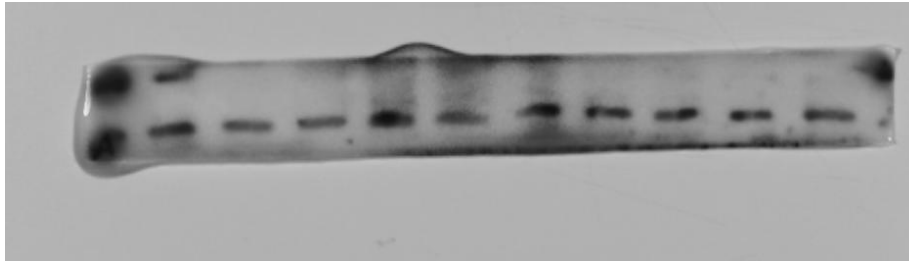

Actin

Supplement: Supplementary file 4 — Additional file 4. WB raw data. [file 13062_2023_401_MOESM4_ESM.pdf]
